# Rapid fusion between mesenchymal stem cells and cardiomyocytes yields electrically active, non-contractile hybrid cells

**DOI:** 10.1038/srep12043

**Published:** 2015-07-10

**Authors:** Ilya Y. Shadrin, Woohyun Yoon, Liqing Li, Neal Shepherd, Nenad Bursac

**Affiliations:** 1 Department of Biomedical Engineering, Duke University, Durham, NC

## Abstract

Cardiac cell therapies involving bone marrow-derived human mesenchymal stem cells (hMSCs) have shown promising results, although their mechanisms of action are still poorly understood. Here, we investigated direct interactions between hMSCs and cardiomyocytes *in vitro*. Using a genetic Ca^2+^ indicator gCaMP3 to efficiently label hMSCs in co-cultures with neonatal rat ventricular myocytes (NRVMs), we determined that 25–40% of hMSCs (from 4 independent donors) acquired periodic Ca^2+^ transients and cardiac markers through spontaneous fusion with NRVMs. Sharp electrode and voltage-clamp recordings in fused cells showed action potential properties and Ca^2+^ current amplitudes in between those of non-fused hMSCs and NRVMs. Time-lapse video-microscopy revealed the first direct evidence of active fusion between hMSCs and NRVMs within several hours of co-culture. Application of blebbistatin, nifedipine or verapamil caused complete and reversible inhibition of fusion, suggesting potential roles for actomyosin bridging and Ca^2+^ channels in the fusion process. Immunostaining for Cx43, Ki67, and sarcomeric α-actinin showed that fused cells remain strongly coupled to surrounding NRVMs, but downregulate sarcomeric structures over time, acquiring a non-proliferative and non-contractile phenotype. Overall, these results describe the phenotype and mechanisms of hybrid cell formation via fusion of hMSCs and cardiomyocytes with potential implications for cardiac cell therapy.

Cell-based therapies hold great promise for restoring the loss of cardiac function in myocardial infarction and heart failure^1^. Human mesenchymal stem cells (hMSCs), in particular, have received much attention owing to their mesodermal multipotency^2^ and ability to secrete pro-angiogenic and anti-apoptotic factors^3^. Numerous preclinical studies applying hMSCs to infarcted myocardium have revealed their positive effects such as reduction in scar size, improved cardiac contractility, and increased tissue perfusion^3^. While it is still unclear how MSCs elicit these effects, proposed mechanisms have included paracrine signaling^4^, differentiation into new cardiomyocytes^5^, fusion with host cardiomyocytes^6^, and activation of endogenous cardiac stem cells^7^. The relative significance of these mechanisms is widely debated, and the exact biomolecular pathways responsible for the therapeutic effects remain to be fully elucidated.

While direct, real-time studies of cardiomyocyte/non-cardiomyocyte interactions *in situ* are very difficult to perform, heterocellular co-culture studies offer a versatile experimental setting to track the fate and function of individual cells *in vitro*. In particular, several previous studies have suggested the existence of mitochondria-containing nanotubular connections between cardiomyocytes (CMs) and endothelial progenitor cells (EPCs)^8^ as well as CMs and MSCs^9^,^10^,^11^, implying that non-CMs may alter CM fates through partial cell fusion and mitochondrial transfer. Spontaneous permanent cell fusion between CMs and non-CMs has been also observed *in vitro*^11^,^12^,^13^,^14^,^15^,^16^,^17^,^18^,^19^ and *in vivo*^6^,^17^,^18^,^20^,^21^,^22^, however the frequency of fusion and the ability of CMs to proliferate or contract post fusion significantly differed in different reports. Furthermore, while the hybrid cells derived through cell fusion have been characterized for the expression of different cardiac markers, functional properties of these cells and mechanisms of hMSC-CM fusion have been studied in much less detail.

In this study, we have utilized co-cultures of hMSCs and neonatal rat ventricular myocytes (NRVMs) to systematically explore the conditions leading to high rate of spontaneous fusion between hMSCs and NRVMs and have characterized the kinetics of this process and electrophysiological properties of the resulting hybrid cells. We demonstrate that the fusion process occurs over a period of hours and leads to the formation of initially contractile cells that progressively lose ordered sarcomeric structures and their ability to contract, and attain electrical properties in between those of passive hMSCs and active NRVMs. The fusion events can be completely and reversibly blocked by pharmacological decoupling of actomyosin bridging and inhibition of Ca^2+^ channels. As such, our studies provide novel insights in the fusion of hMSCs and native cardiomyocytes with potential implications for current cell-based therapies.

## Methods

### Cell preparation

This study conforms to the Guide for the Care and Use of Laboratory Animals published by the US National Institutes of Health (NIH Publication #85-23, revised 1996). All animals were treated according to protocols approved by the Duke University Institutional Animal Care and Use Committee (IACUC), protocol #A165-12-06. Neonatal Sprague-Dawley rats were euthanized via decapitation and ventricular cells were enzymatically isolated using trypsin and collagenase, as previously described^23^,^24^. Faster-attaching fibroblasts were removed using two 1 hr differential preplating steps. The remaining cells, mostly cardiomyocytes (NRVMs), were plated on fibronectin coated (15 ug/mL, Sigma) 35 mm μ-dishes (iBidi) at a density of 0.18 × 10^6^/400 uL in a 10% fetal bovine serum/10% horse serum (Gibco) cardiac medium (containing high glucose (4.5 g/L) DMEM, 100 U/mL penicillin, and 100 ug/mL streptomycin (Gibco)). To efficiently label NRVMs, cells were transduced with reporter lentiviruses at the time of seeding in the presence of 8 ug/mL polybrene (Millipore). The next day, NRVMs were rinsed and treated with 10 ug/mL mitomycin C (Sigma) for 3 hrs to prevent cardiac fibroblast overgrowth, and 24 hrs later, switched to cardiac media containing 2% horse serum (HS). Media was changed every 2 days thereafter.

Frozen vials of hMSCs from the bone marrow of 4 healthy donors were purchased from either Lonza (PT-2501) or Dr. Darwin Prockop (Tulane Center for the Preparation and Distribution of Adult Stem Cells), thawed and expanded in MSC basal medium (MSCBM, Lonza). Cells were characterized by the vendor for their ability to differentiate into osteogenic, chondrogenic, and adipogenic lineages, and immunoreactivity for CD105, CD166, CD29, and CD44, but not CD14, CD34 or CD45. hMSCs were transduced with lentiviruses overnight (in the presence of 8 ug/mL polybrene), and passaged using 0.05% trypsin-EDTA (Gibco) every 5–7 days at 80–90% confluence for several passages prior to initiation of co-cultures to remove any risk of secondary transduction^25^. hMSCs from passages 8–14 were used for co-culture studies.

### Studies of cell fusion in hMSC- NRVM co-cultures

All co-cultures were plated at low density to produce a monolayer of cells, minimizing the likelihood of two cells co-localizing on top of each other. Specifically, lentivirally labeled hMSCs suspended in MSCBM were added to NRVMs 5 days after NRVM plating (e.g. 4.5 × 10^3^ hMSCs for 1:40 hMSC:NRVM ratio), and switched to 2% HS cardiac media the following day. Reverse plated co-cultures were generated by transducing NRVMs with mCherry lentivirus and 8 μg/mL polybrene in an overnight suspension and adding on top of hMSC-gCaMP3 subcultures. Percent of Ca-oscillating cells was assessed by counting number of gCaMP3-flashing cells out of 100 gCaMP3^+^ hMSCs in ~20 random fields of view through a 20x objective. For dually labeled co-cultures, percent fusion was quantified as the number of double-positive (mCherry^+^/gCaMP3^+^) cells out of 100 gCaMP3^+^ hMSCs. For co-culture studies using the Cre-Lox expression system, percent fusion was measured as the number of GFP^+^ hMSCs out of 100 GFP^+^ or mCherry^+^ hMSCs. Measurements were performed at different time points between 1 hr and 7 days after addition of hMSCs.

For pharmacological studies in hMSC-NRVM co-cultures, the following drugs were added at the time of hMSC plating and removed after 24 hrs of co-culture: DMSO, blebbistatin, nifedipine, verapamil hydrochloride, N-benzyl-p-toluene sulphonamide (BTS), 2,3-butanedione monoxime (BDM), methyl-β-cyclodextrin (MBCD), gadolinium III chloride, penicillin-streptomycin, isoproterenol hydrochloride (dissolved in water with ascorbic acid), phenylephrine hydrochloride, cytochalasin D, CT04 (Rho inhibitor, Cytoskeleton), Y27632 (ROCK inhibitor, Millipore), and EDTA (Invitrogen, stock). Unless otherwise noted, all drugs were purchased from Sigma and dissolved in DMSO.

### Electrophysiology

For the measurement of transmembrane potential, hMSCs were co-cultured with NRVMs as described above and superfused with 35 °C modified Tyrode’s solution containing (in mM): NaCl, 135; KCl, 5.4; CaCl_2_, 1.8; NaH_2_PO_4_, 0.33; Hepes, 5; Glucose, 5; pH 7.4. Cells were selected to be well adhered to the dish with no other underlying cells and placed into the 16 μm × 16 μm field of view of the PMT (DeltaRam V, PTI, Monmouth Junction, NJ). A sharp-tipped 3M KCl-filled microelectrode (R~50 MΩ), connected to the head stage of the recording amplifier in zero-current mode (200B clamp amplifier and Digidata 1300 board, Axon Instruments, Burlingame, CA), was positioned over the cell under direct vision and advanced slowly until the appearance of the full membrane potential and action potentials. Electrical signals were recorded at 4 kHz after filtering at 2 kHz. GFP fluorescence was recorded simultaneously with a photomultiplier tube, using broadband excitation and a FITC filter. The output of the photomultiplier was digitized at 100 fps with Felix32 software (PTI). All data were analyzed using Origin software (OriginLab Corp., Northhampton, MA).

### Time-lapse video microscopy

Co-cultures of hMSC/NRVM were prepared on FN-coated 35 mm Fluorodishes (World Precision Instruments) as described. Four hours after hMSCs were added to NRVMs, initial plating media was replaced with fresh cardiac media and Fluorodishes were mounted on an opto-electronically encoded XY stage of a Zeiss Axio Observed Z1 inside a Pecol XL S1 incubator. Twenty locations where hMSCs were found in close proximity with NRVMs were selected per dish, and 3-channel (red, green, DIC) images were taken at each location every 2.5 min using a high-resolution CCD camera and automated stage control through MetaMorph software (Molecular Devices). Co-cultures were imaged for 3.5 – 4.5 hrs, and resulting images were compiled into time-lapse movies using MetaMorph.

### Real-time cell motion analysis

For cellular motility studies, hMSC nuclei were manually tracked in MetaMorph Premier (Molecular Devices), and a custom Matlab (Mathworks) program was used to fit motility data to a Persistent Random Walk model^26^:


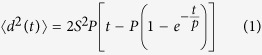


In equation (1), 〈*d*^*2*^(*t*)〉 represents the mean-squared displacement (MSD) of a cell calculated using overlap methods, S is the speed of motion, and P is the persistence time, the characteristic time that a cell moves in one direction before switching to another direction. The mean free path, representing the characteristic length that a cell moves during time P, can be calculated as the product of S and P. Per other reports analyzing hMSC motility^27^, cells with a mean free path greater than 3 μm were considered migratory and used for calculating population averages of S, P and S*P, while cells with speed values over 1.4 μm/min were considered outliers and not included in analysis. For motility assays testing nifedipine or blebbistatin, drugs were added to the culture at the time of hMSC plating. Specifically for blebbistatin, hMSCs were labeled with mCherry while NRVMs remained unlabeled, as the use of blue light for imaging of gCaMP3 leads to rapid photo-inactivation of blebbistatin^28^. For each condition, the MSD was calculated by averaging the individual cell MSDs over all possible 52.5 min periods during the entire time-lapse video. Distributions of real-time motion analysis parameters (S, P, S*P) were determined to be non-parametric using Shapiro-Wilks test and subsequently analyzed using a Kruskal-Wallis (Rank Sum) test followed by Wilcoxon pair testing.

### Statistics

Experimental data reported as mean ± SEM was compared using Student’s t-test or one-way ANOVA followed by Tukey’s post-hoc test. All analysis was done using JMP Pro 11 software (SAS Institute). A p-value of 0.05 was considered significant.

*Additional materials and methods are described in online Supplemental Materials.*

## Results

### hMSC-EGFP express cardiac markers and activate human cardiac genes in co-culture with NRVMs

**Figure 1:**
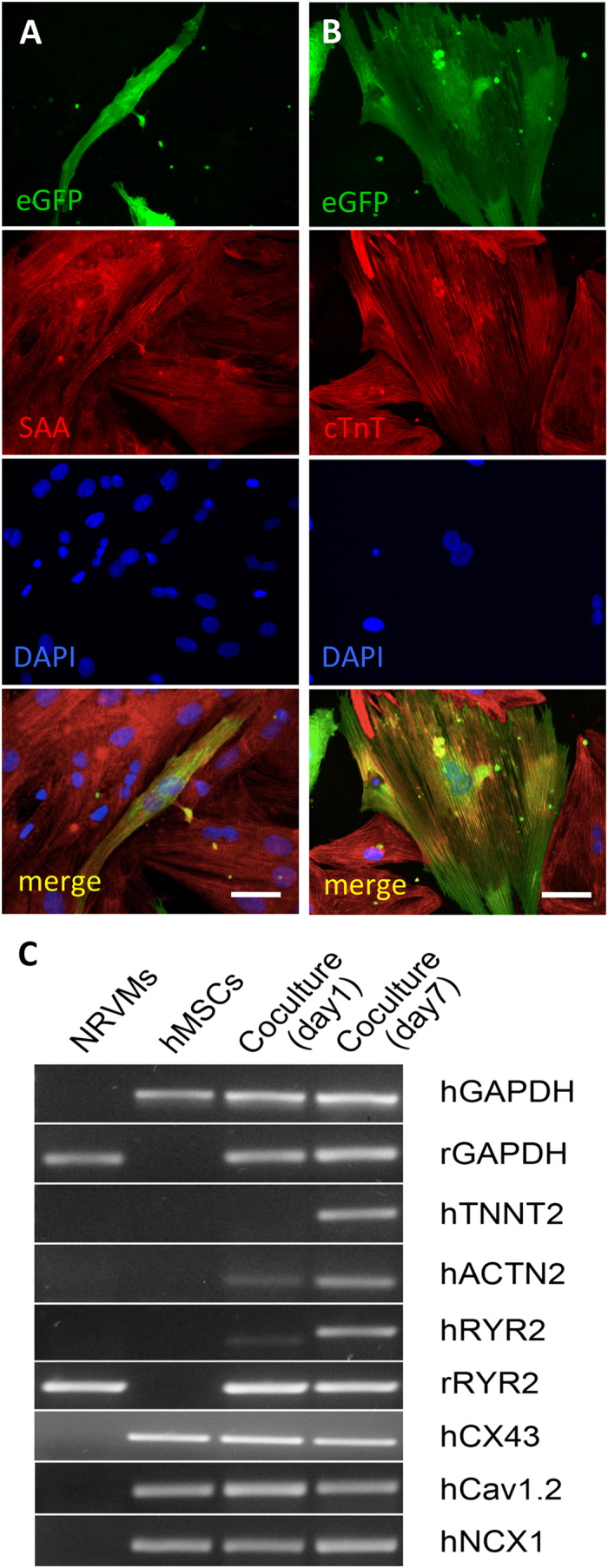
hMSCs in co-culture with NRVMs express cardiac markers and activate human cardiac genes. **A**,**B**) Representative confocal images of immunostaining for sarcomeric α-actinin (SAA) and cardiac troponin T (cTnT) from 7-day old hMSC-eGFP/NRVM co-cultures showing eGFP^+^/α-actinin^+^ (A) and eGFP^+^/cTnT^+^ (B) cells. (**C**) RT-PCR using rat- and human-specific primers shows activation of human cardiac genes (hTNNT2, hACTN2, hRYR2) in hMSC-eGFP/NRVM co-culture. Scale bar 50 μm.

To study the direct interactions between hMSCs and NRVMs, hMSCs were transduced with a lentivirus encoding eGFP and added to a subconfluent monolayer of NRVMs. After 7 days of co-culture, immunostaining for sarcomeric α-actinin (SAA) and cardiac troponin T (cTnT) showed the presence of eGFP^+^/SAA^+^ (Fig 1A) and eGFP^+^/cTnT^+^ (Fig. 1B) double-positive hMSCs. To determine the effect of co-culture on cardiac gene expression, an RT-PCR was performed using rat- and human-specific primers (Supplementary Table 1). In contrast to NRVMs or hMSCs alone, co-culture of hMSCs/NRVMs showed a time-dependent activation of human cardiac genes, including those encoding cTnT (hTNNT2), α-actinin (hACTN2), and ryanodine receptor (hRYR2) (Fig. 1C). This data suggested the potential of hMSCs to acquire cardiac fate in co-cultures with NRVMs.

### hMSC-gCaMP3 in co-culture with NRVMs exhibit robust Ca^2+^ transients preceded by membrane voltage depolarizations

**Figure 2:**
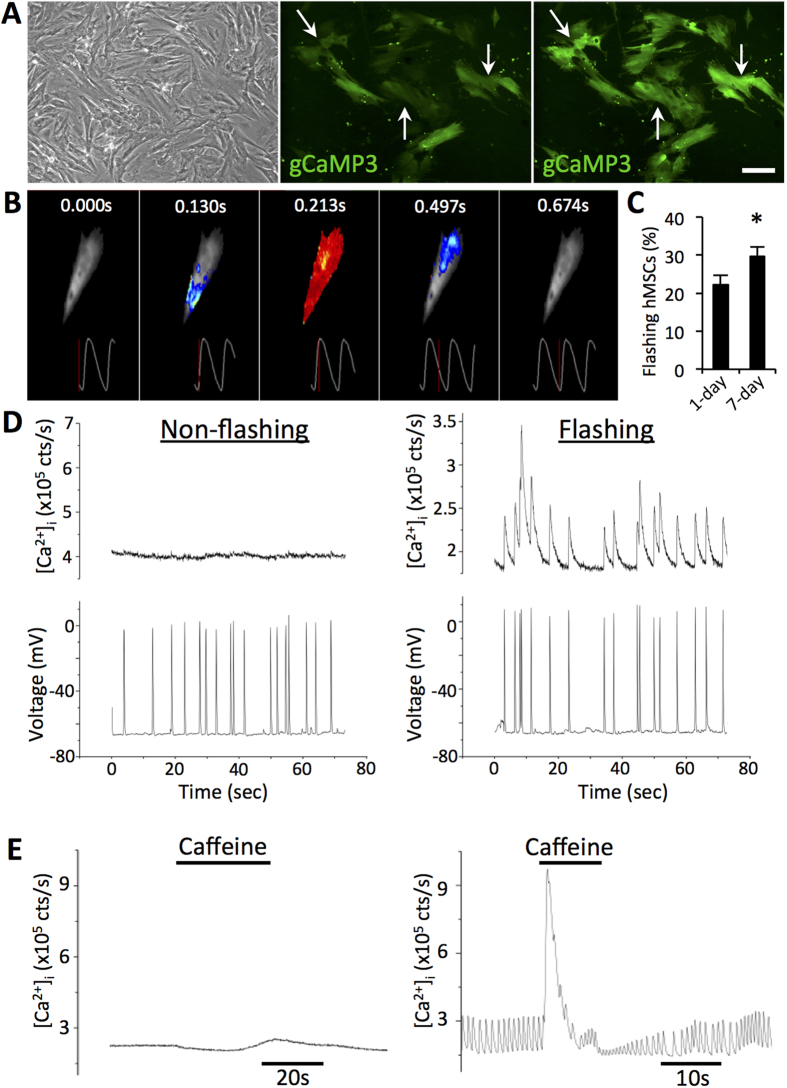
hMSCs-gCaMP3 in co-culture with NRVMs exhibit both membrane voltage fluctuations and Ca^2+^ transients. **A**) In co-cultures of hMSCs and NRVMs (phase contrast, top left), hMSCs transduced with Ca^2+^-sensitive indicator gCaMP3 exhibit flashes of gCaMP3 (examples shown by arrows) representing Ca^2+^ transients occurring in synchrony with NRVM contractions. Scale bar 100 μm. (**B**) Time snapshots during optically recorded gCaMP3 Ca^2+^ transient shown by pseudo-colored fluorescence intensity and corresponding time traces (below). C) Quantification of the percent of flashing hMSCs at 1 and 7 days of co-culture (n = 12 co-cultures, *p < 0.05, Student’s t-test). D) Representative sharp electrode recordings from 1 day-old hMSC-gCaMP3/NRVM co-cultures show that both flashing and non-flashing hMSCs have membrane voltage oscillations (bottom), but only flashing cells demonstrate synchronous gCaMP3 (Ca^2+^) transients (top). E) Unlike non-flashing hMSCs (left), flashing hMSCs from 1-day co-cultures demonstrate sensitivity to local superfusion with 10 mM caffeine (right).

Given the importance of Ca^2+^ handling in cardiomyocytes, we sought to better understand the Ca^2+^ handling properties of hMSCs in co-culture with NRVMs. We transduced hMSCs with a high-titer lentivirus encoding a genetic sensor of intracellular Ca^2+^ concentration, gCaMP3^29^, and cultured the transduced hMSCs on top of NRVM monolayers (Fig. 2A, top left). After one day in co-culture, live-cell imaging showed that a significant portion of the hMSC-gCaMP3 exhibited fluorescent flashes (Fig. 2A,B; Supplementary Video 1) in synchrony with NRVM contractions. Quantification of live-cell co-cultures revealed that fraction of transduced hMSCs that exhibited gCaMP3 flashes increased from 22.1 ± 2.5% at 1 day of co-culture to 29.6 ± 2.5% after 7 days (Fig. 2C). Varying the initial hMSC:NRVM seeding ratio from 1:5 to 1:100 revealed a dose-dependent increase in the percentage of flashing hMSCs at lower numbers of added hMSCs (Supplementary Fig. S1). A seeding ratio of 1:40 offered a balance between sufficient hMSC numbers for various assessments and ease of distinguishing between individual cells, and was thus used for all subsequent experiments. Tracking of individual flashing hMSCs in 7-day co-cultures followed by fixation and immunostaining showed that 95.3 ± 2.1% and 88.4 ± 2.6% of flashing hMSCs stained positive for SAA and cTnT, respectively (Supplementary Fig. S1), indicating that the vast majority of flashing cells expressed cardiac-specific proteins.

Sharp electrode recordings from 1d-old co-cultures demonstrated membrane voltage fluctuations in both flashing and non-flashing hMSCs (Fig. 2D, bottom), but only flashing hMSCs exhibited synchronous Ca^2+^ oscillations (Fig. 2D, top) following each voltage spike. In contrast to their non-flashing counterparts, flashing hMSCs demonstrated sensitivity to caffeine, an allosteric activator of the ryanodine receptor (Fig. 2E), indicative of at least partially functional intracellular Ca^2+^ machinery.

### Flashing hMSCs arise from fusion between hMSCs and NRVMs

**Figure 3:**
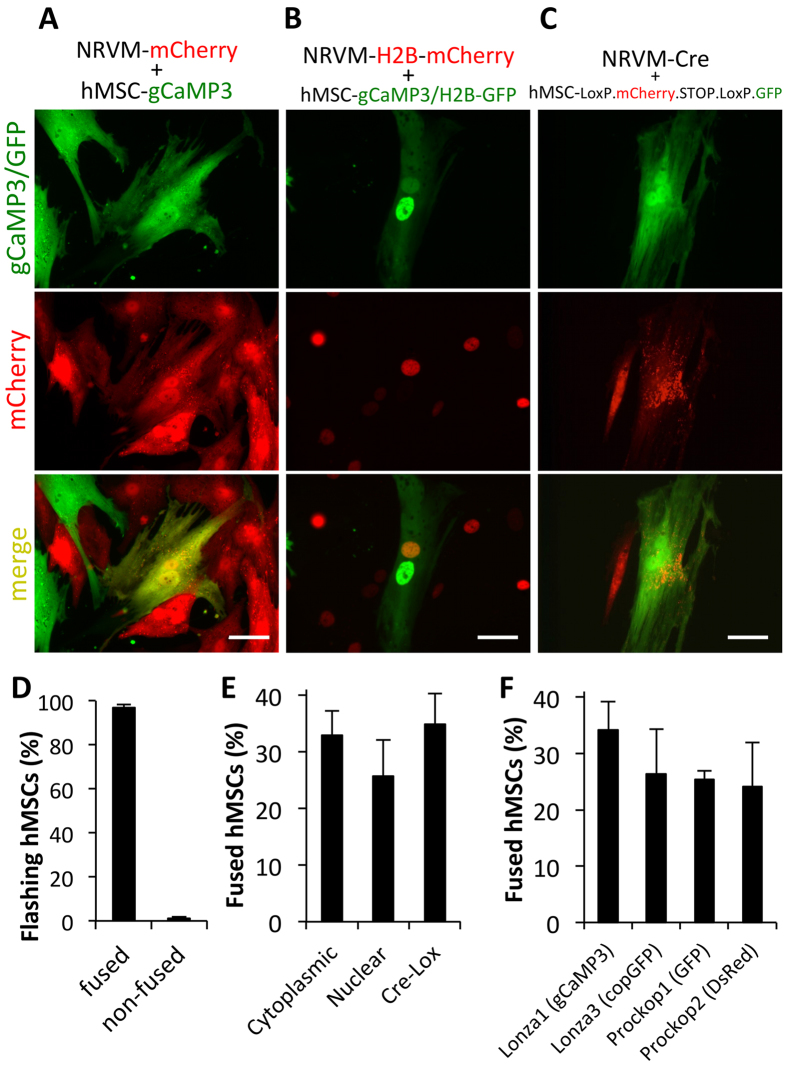
Flashing hMSCs arise from fusion between hMSCs and NRVMs. **A**) Representative images from 1-day co-cultures of NRVM-mCherry with hMSC-gCaMP3 with evidence of dually-labeled fused cells (arrows). (**B**) Nuclear labeling of 1-day co-culture of NRVM-H2B-mCherry and hMSC-gCaMP3/H2B-GFP shows both NRVM and hMSC nuclei in fused cells. (**C**) 1-day co-culture of NRVM-Cre with hMSC-LoxP.mCherry.STOP.LoxP.GFP shows Cre-mediated switching of hMSC labeling from mCherry to GFP, indicative of fusion with Cre-expressing NRVMs. All scale bars 50 μm. (**D**) Quantification of flashing hMSCs shows that nearly 100% of flashing hMSCs have markers of fused cells and virtually no non-fused hMSCs show flashing (n = 10 co-cultures). (**E**) These 3 independent methods to assess and quantify cell fusion all show that ~30% of hMSCs undergo fusion after 7 days of co-culture with NRVMs (nuclear: n = 2; cytoplasmic: n = 6; Cre-lox: n = 9 co-cultures). (**F**) Similar degree of fusion has been measured (by Cre-lox method) for hMSCs from 4 independent donors (n = 2–5 co-cultures).

**Figure 4:**
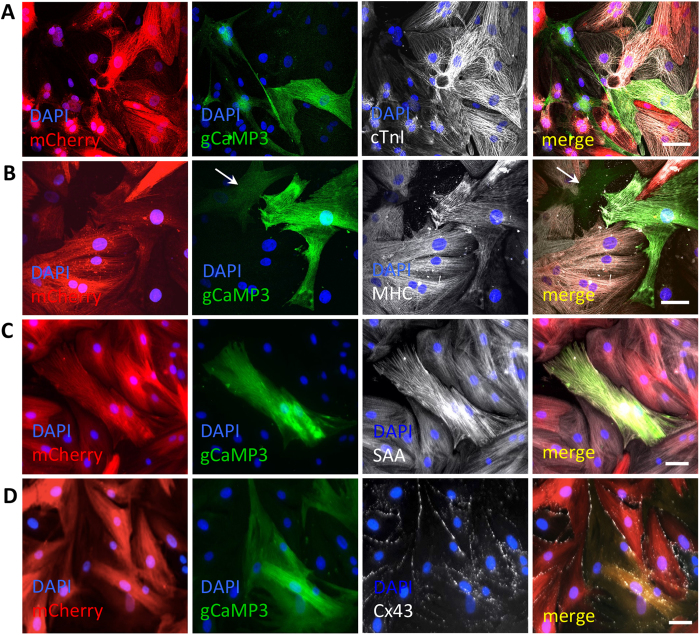
Fused cells express various cardiac markers and couple to NRVMs. **A**–**D**) Representative images of 7-day old hMSC-gCaMP3/NRVM-mCherry co-cultures showing that dually labeled fused cells express various cardiac markers including cardiac troponin I (cTnI, A), myosin heavy chain (MHC, B), sarcomeric α-actinin (SAA, C), and gap junctional protein Connexin-43 (Cx43, D). A non-fused hMSC is shown with arrow in B. All scale bars 50 μm.

Since cardiac differentiation from stem cells typically requires at least 7 days^30^, the presence of functional Ca^2+^ machinery in hMSCs after only 1 day in co-culture suggested a potential for cell fusion. To test this hypothesis, hMSCs transduced with gCaMP3 were co-cultured with NRVMs previously transduced with a mCherry lentivirus. After 1 day of co-culture, live-cell imaging revealed the presence of mCherry^+^/gCaMP3^+^ double positive cells, which we termed hybrid cells owing to the apparent exchange of cytoplasmic contents (Fig. 3A). Quantification of flashing hMSCs vs. hybrid cells showed that 96.7 ± 1.52% of hybrid cells and only 1.3 ± 0.63% of gCaMP3^+^/mCherry^-^ cells exhibited gCaMP3 flashes (Fig. 4D). Co-cultures set up with a reversed plating order (hMSCs first, NRVM second), reverse labeling order (hMSC-mCherry, NRVM-gCaMP3), or with unlabeled NRVMs still produced hybrid cells at similar frequencies (Supplementary Figs. S2,S9). Importantly, no phagocytosis was seen in hMSCs or NRVMs in co-cultures with added NRVM or hMSC lysates, respectively (Supplementary Fig. S3), further suggesting occurrence of cell fusion. Additionally, no dually labeled cells were found in hMSC/hMSC, NRVM/NRVM, or hMSC/neonatal rat ventricular fibroblast co-cultures (Supplementary Fig. S4), indicating that formation of hybrid cells was specific to the hMSC/NRVM interaction. Finally, hybrid cells were observed when NRVMs were co-cultured with rat MSCs and when hMSC were co-cultured with mouse ESC-derived cardiomyocytes (mESC-CMs) (Supplementary Fig. S5), indicating robustness of this MSC/CM interaction across various species.

Since exchange of cytoplasmic contents can occur via both transient and permanent intercellular connections, we aimed to confirm the formation of fused cells using more rigorous assessments. We performed nuclear labeling of hMSCs and NRVMs using lentiviruses encoding gCaMP3/H2B-GFP and H2B-mCherry, respectively. Live-cell imaging of 1d-old co-cultures revealed flashing cells that contained both hMSC and NRVM nuclei (Fig. 3B). To definitively confirm cell fusion, we utilized a Cre-Lox recombination system wherein Cre recombinase can excise DNA fragments floxed by LoxP sites. Using this approach, hMSCs were transduced with a lentivirus encoding a LoxP.mCherry.STOP.LoxP.GFP cassette and co-cultured with NRVMs transduced with a lentivirus encoding Cre recombinase. After 1 day of co-culture, live-cell imaging revealed the presence of GFP-expressing hMSCs, confirming fusion with the NRVMs (Fig. 3C). Quantification of hybrid cells in 7d co-cultures by cytoplasmic labeling, nuclear labeling, or Cre-Lox recombination consistently showed that 25–35% of hMSCs were fused (Fig. 3E). To rule out a potential artifact from using a particular hMSC line, the high level of fusion was confirmed using Cre-Lox system and hMSCs derived from four independent donors (Fig. 3F).

### Fused cells express cardiac markers, are coupled to NRVMs, and do not proliferate

To further assess the cardiomyocyte-like features of the fused cells, 7d hMSC-gCaMP3/NRVM-mCherry co-cultures were immunostained for multiple cardiac markers. Consistently across all co-cultures, fused cells expressed cardiac troponin I (cTnI), SAA, and myosin heavy chain (MHC, Fig. 4A–C), and were robustly electrically and mechanically coupled to nearby NRVMs via the gap-junction protein Cx-43 (Fig. 4D) and adherens junction protein N-Cadherin (Supplementary Fig. S6), respectively. In contrast, non-fused (gCaMP3^+^/mCherry^-^) hMSCs did not express such markers (Fig. 4B, Supplementary Fig. S7) and exhibited limited Cx43 expression (Supplementary Fig. S7), consistent with previous reports of poor gap junctional coupling in wild-type adult hMSCs^31^. Quantification of the cell nuclear number with time of co-culture (Supplementary Fig. S8) revealed that the majority of fused cells contained 2 or 3 nuclei (of which 1 always originated from the hMSC and 1 or more originated from the NRVMs), and that mononucleated synkaryons were rare and did not appear until 24 hrs into the co-culture. Immunostaining for the mitotic marker Ki67 failed to identify any Ki67^+^ fused cells (Supplementary Fig. S8), indicating that fused cells were primarily non-proliferative heterokaryons.

### Fused cells show electrophysiological properties in between those of hMSCs and NRVMs

**Figure 5:**
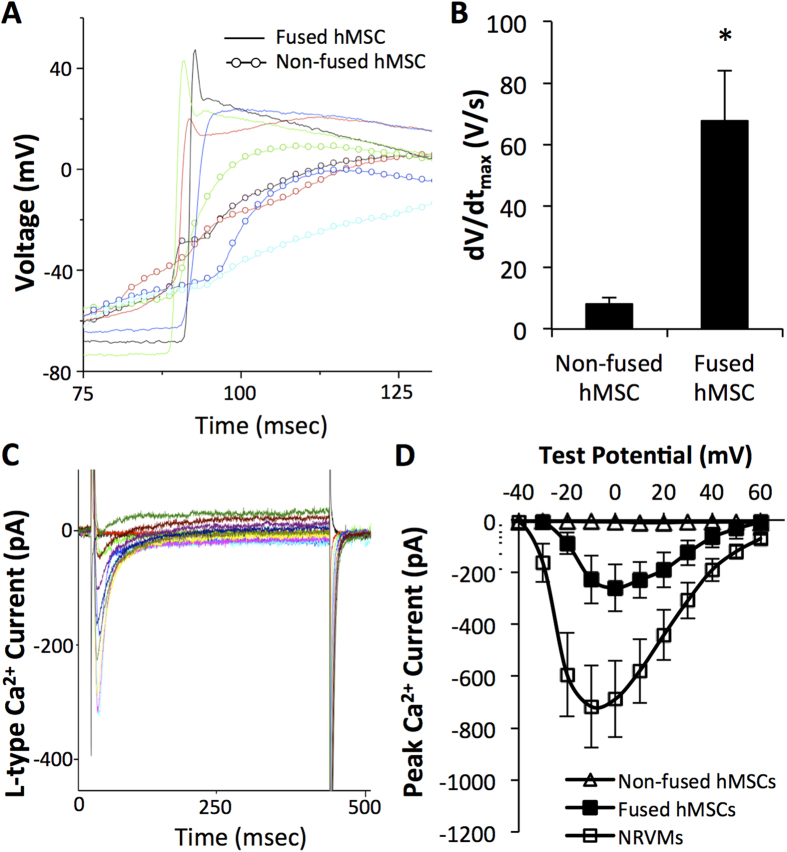
Fused cells have electrophysiological properties in between hMSCs and NRVMs. **A**) Representative traces of action potential depolarization in fused green cells (lines) and red non-fused hMSCs (circles and lines) in 1-day hMSC-LoxP.mCherry.STOP.LoxP.GFP/NRVM-Cre co-cultures. (**B**) Depolarization upstroke velocity (dV/dt_max_) in fused cells and non-fused hMSCs; n = 5 cells each, *p < 0.02, Student’s t-test. (**C**) Representative example of whole-cell voltage clamp recording of GFP^+^ cell dissociated from 7-day co-culture of NRVM-Cre and hMSC-LoxP.mCherry.STOP.LoxP.GFP (i.e. fused cells) shows robust inward Ca^2+^ currents. (**D**) Average current-voltage relationships from freshly dissociated red non-fused hMSCs, green fused cells, and NRVMs from 7-day co-cultures show that total L-type Ca^2+^ currents in fused cells are in between those of non-fused hMSCs and NRVMs; n = 5–9 cells per group.

Sharp electrode recordings in 1-day co-cultures (Fig. 5A) further revealed that fused cells had a significantly faster membrane voltage upstroke (dV/dt_max_ of 67.6 ± 16.01 V/s) relative to non-fused hMSCs (8.2 ± 2.24 V/s, Fig. 5B), though still less than the typical values we previously reported for NRVMs (105.9 ± 9.5 V/s^32^). Similarly, fused hMSCs showed a trend towards lower resting potentials (−66 ± 2 mV) compared to non-fused hMSCs (−63 ± 1 mV), but still higher than we reported for NRVMs (−70.1 ± 1.2 mV^32^). Whole-cell voltage clamp recordings in dissociated cells from 7d-old co-cultures showed that fused cells (cell capacitance C = 129 ± 21 pF) were significantly larger than non-fused hMSCs (C = 46 ± 5 pF) or NRVMs (C = 65 ± 10 pF). The fused cells showed inward L-type Ca^2+^ currents (Fig. 5C) with the maximum total current and current density (−260.7 ± 89.8 pA, −2.6 ± 1.1 pA/pF, Fig. 5D, filled squares) that were higher than those of non-fused hMSCs (−9.7 ± 3.2 pA, −0.3 ± 0.2 pA/pF, open triangles) but still lower than those of NRVMs (−718.0 ± 157.6 pA, −10.3 ± 1.5 pA/pF, open squares). Together, this data indicated that fusion of hMSCs with NRVMs generated hybrid cells capable of electrical coupling and electrophysiological properties intermediate between those of hMSCs and NRVMs.

### hMSC-NRVM fusion occurs early in co-culture and leads to loss of sarcomeric structures and contractions

**Figure 6:**
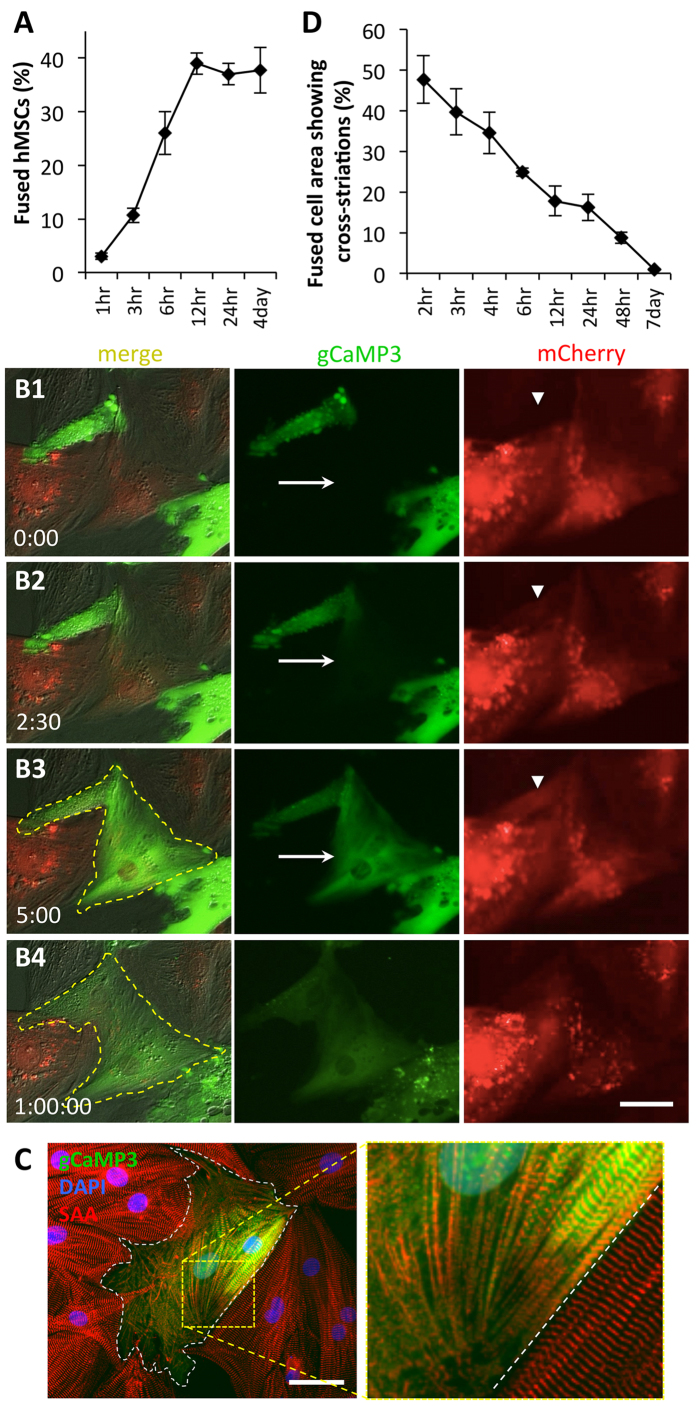
Fusion of hMSCs with NRVMs occurs rapidly and leads to loss of sarcomeric structures. **A**) Percent of fused cells increases with time of hMSC/NRVM co-culture, reaching a steady-state after only 12 hrs (4day: n = 8 co-cultures; all others: n = 2–3 co-cultures). (**B**1–4) Representative snapshots from a live-cell time-lapse video of hMSCs-gCaMP3/NRVM-mCherry co-culture. Note rapid spread of gCaMP3 label from hMSC to NRVM (arrow in B2 and B3) and occurrence of mCherry in hMSC (arrowhead in B2 and B3). Within 1 hr, membrane contour of the fused cell (yellow dashed line) significantly changes (B4). Elapsed time in minutes shown at bottom left. (**C**) Representative immunostaining of sarcomeric α-actinin (SAA, red) after 6 hrs of hMSC-gCaMP3/NRVM co-culture showing breakdown of sarcomeres in fused cell (white dashes – border of hybrid cell, roughly 25% of which has intact striations). (**D**) Percent area of a fused cell covered by cross-striations decreases over time, indicating loss of sarcomeres; n = 4–10 cells per time point. All scale bars 50 μm.

Given the significant emergence of fused cells in 24 hrs of co-culture, we decided to quantify the kinetics of the fusion process. We found that fusion begins as early as 1hr after seeding of hMSCs and reaches steady state after 12 hrs (Fig. 6A). Since fusion percentage steadily increased in early co-culture, we performed time-lapse video microscopy on freshly seeded hMSC-gCaMP3/NRVM-mCherry co-cultures to visualize the fusion events. As shown in the representative video (Supplementary Video 2), an hMSC began flashing gCaMP3 shortly after fusing with a neighboring NRVM. Fusion resulted in rapid (within 5 minutes) spread of gCaMP3 from hMSC into the entire NRVM (Fig. 6B1–3, arrows) and mCherry from NRVM into the entire hMSC (Fig. 6B1–3, arrowheads), which was followed by a change in the membrane contour of the fused cell over 1 hour (Fig. 6B3–4, dashed line).

When imaging fused cells at various time points, we noticed that early (first few hours, Supplementary Videos 2,3) fused cells retained a contractile phenotype while most late (>1d old) fused cells no longer contracted (Supplementary Video 4). We thus performed immunostaining of sarcomeric α-actinin (SAA) at various time points of co-culture to assess whether changes in sarcomeric organization in fused cells could explain the gradual disappearance of contractions despite the presence of robust Ca^2+^ transients detected by gCaMP3. A representative image of a fused cell in a 6 hr co-culture shows an interface region between fusing hMSC and NRVM where parallel registration of SAA^+^ sarcomeres was being lost (Fig. 6C). Quantification of the percent area of a fused cell containing intact cross-striations showed a steady decrease in organized sarcomeric structures over time, with >90% of striations disappearing after 2 days of co-culture (Fig. 6D) and entirely absent in 7d-old cultures (Supplementary Fig. S9).

### Reversible inhibition of fusion via direct Myosin II and L-type Ca^2+^ channel blockade

**Figure 7:**
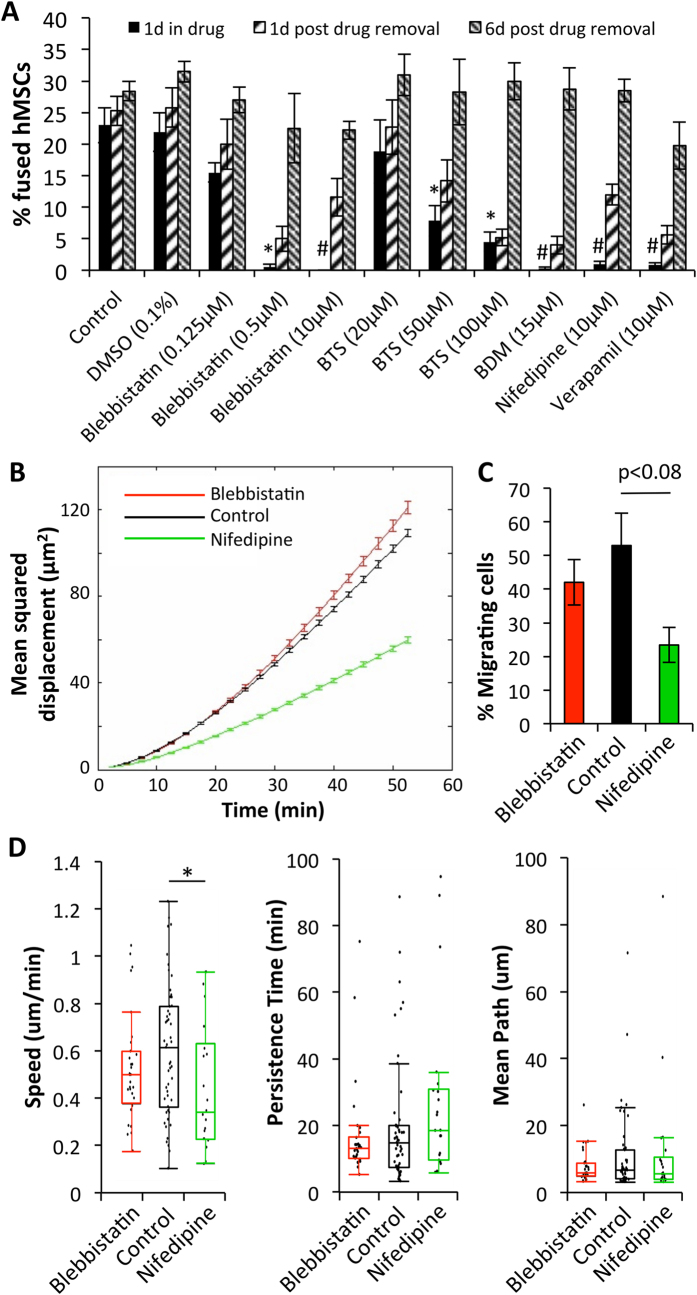
Reversible inhibition of fusion via Myosin II and Ca^2+^ channel blockade. **A**) Fraction of fused hMSCs in 1, 2, and 7-day co-cultures after application of various drugs. Drugs were added at the start of co-culture and removed after 1 day; n = 3–15 co-cultures; *p < 0.01, #p < 0.0001 relative to “Control, 1d in drug”, ANOVA with Tukey’s post-hoc test. (**B**) Mean squared displacement of hMSCs in co-culture with NRVMs during a 3.5 hr time-lapse imaging in the presence of various drugs. (**C**) Fraction of migrating cells across all co-cultures. (**D**) Parameter fitting from individual cells for speed, persistence time and mean path of migration; *p < 0.05. (**B**–**D**) n = 2–3 co-cultures, N = 25–54 cells per timelapse of co-culture.

To investigate the mechanism of hMSC/NRVM fusion, we assessed percentage of fused cells following a 24 hr drug application at the time of hMSC seeding onto the NRVMs. We found that treatment with myosin II inhibitors (10 μM blebbistatin, 100 μM BTS, or 15 mM BDM) or L-type Ca^2+^ channel blockers (10 μM nifedipine or 10 μM verapamil) significantly reduced percent of fused hMSCs (in average 1.4 ± 0.43% in treated vs. 23 ± 2.76% in control, Fig. 7A, Supplementary Fig. S10). Following removal of these drugs after 24 hrs, fusion events were gradually restored by 7 days of co-culture to control levels (in average 25 ± 1.51% vs. 28.43 ± 1.52% in control, Fig. 7A), indicating reversibility of the fusion blockade. Interestingly, no effect on fusion was observed with application of numerous other drugs, including DMSO (vehicle for blebbistatin, BTS, nifedipine and verapamil), methyl-beta cyclodextrin, gadolinium, streptomycin, phenylephrine, and isoproterenol, while cytochalasin-D prevented spreading of hMSCs and caused rounding of NRVMs (Supplementary Fig. S10). Additionally, hMSCs treated with either blebbistatin or nifedipine for 24 hrs prior to addition to NRVMs still fused similar to untreated cells (not shown), suggesting a role of these drugs during the actual process of fusion.

Finally, we assessed whether fusion-inhibiting drugs affected cell motility as a means of preventing cell-cell interactions leading to fusion by performing a 3.5 hr time-lapse imaging of early co-cultures in the presence or absence of blebbistatin and nifedipine. Consistent with previous reports^33^,^34^, nuclear tracking revealed a decrease in mean square displacement and migration speed of hMSCs in nifedipine but not blebbistatin-treated co-cultures (Fig. 7B–D), which along with the evidence of fusion in minimally motile hMSCs (Supplementary Videos 2,5) suggested limited involvement of cell motility in the process of fusion and implied a more direct mechanism for the drug-mediated inhibition of fusion.

## Discussion

In this study, we describe the formation and characterization of cardiac-like hybrid cells generated via complete fusion of hMSCs and NRVMs *in vitro*. In particular, we for the first time show that at relatively low hMSC:NRVM ratios, the two cell types spontaneously and rapidly fuse within hours of co-culture yielding electrophysiologically active hybrid cells that strongly couple to surrounding NRVMs, oscillate membrane potentials, and generate Ca^2+^ transients via functional L-type Ca^2+^ channels and intracellular Ca^2+^ stores. The process of fusion involves rapid exchange of cytoplasmic content between hMSCs and NRVMs but no merger of nuclear content, with virtually all hybrid cells becoming non-proliferative heterokaryons (>95% having 2 or more nuclei). We further show that the fusion process occurs in about one third of hMSCs regardless of donor, is species independent and specific to interaction of MSCs and CMs. While hybrid cells activate human cardiac genes and remain electromechanically coupled to NRVMs and able to cycle Ca^2+^ levels, their sarcomeric structure and contractility are gradually lost, thus implying limited direct benefits to cardiac contractile function *in vivo*.

In the continuing debate over the effects of MSC implantation in the heart^4^,^5^,^6^,^7^, our data provides strong support for the role of spontaneous cell fusion in hMSC-cardiomyocyte interactions. To unambiguously demonstrate the existence of cell fusion in live hMSC-NRVM co-cultures, we applied three independent lentiviral approaches to identify the fused cells, which all yielded consistent quantitative results (Fig. 3E). First, we labeled hMSCs with real-time Ca^2+^-sensing molecule gCaMP3^35^, which allowed us to both assess intracellular Ca^2+^ dynamics in fused cells relative to NRVM contractions as well as monitor the acquisition of Ca^2+^ transients in newly fused hMSCs. Furthermore, lentiviral labeling of hMSC and NRVM nuclei using H2B-fused fluorescent reporters confirmed the cell fusion and allowed us to quantify the origin and number of nuclei in multinucleated hybrid cells, which predominantly contained one hMSC nucleus and 1 or more NRVM nuclei (Supplementary Fig. S8). This also implied the capacity of hMSCs to fuse with multiple NRVMs, which in our cultures are mostly mononuclear. As synkaryons, but not heterokaryons, have been previously shown to be capable of proliferation^36^, the observed scarcity of fused synkaryons in our study may explain lack of Ki67 expression and the apparent non-proliferative phenotype of fused cells. Finally, the use of lentiviral Cre-Lox recombination system served to most rigorously demonstrate hMSC-NRVM fusion, where in the absence of phagocytosis (Supplementary Fig. S3), stable transition of red (mCherry) to green (GFP) label within an hMSC could only arise through a cell fusion with Cre-recombinase expressing NRVMs.

The cell fusion in our study was a spontaneous event, not induced by exogenous fusogens such as vesicular stomatitis virus (VSV-G)^37^, polyethylene glycol^38^, or hemagglutinating virus of Japan (HJV)^38^,^39^. Our measured hMSC fusion percentages of 25–40% fall at the high end the variable range of MSC/CM fusion efficiencies (0.01–40%) reported in other studies^10^,^11^,^12^,^13^,^15^,^17^,^18^,^19^,^40^,^41^, although a majority of these studies investigated co-cultures of rodent MSCs and CMs^11^,^13^,^15^,^17^,^18^,^19^,^40^. The use of time-lapse imaging in conjunction with gCaMP3 labeling allowed us to for the first time directly demonstrate and study kinetics of the fusion process between hMSCs and NRVMs. Interestingly, we found that first fusion events occurred as early as 30 min of co-culture and that only few minutes were sufficient for the exchange of green (gCaMP3) and red (mCherry) cytoplasmic labels between the fused cells (Fig. 6B). Importantly, it took only 2–3 hrs for gCaMP3 flashing to gradually spread from the NRVM side to the entire hMSC part of the fused cell (Supplementary Videos 2 and 5) suggesting that NRVM (rather than newly formed hMSC) Ca^2+^ channels that trafficked and docked to the hMSC membrane were the most likely contributors to the early Ca^2+^ oscillations. Most fusion events occurred within the first 12 hrs of co-culture (Fig. 6A), which was followed by the gradual activation of human cardiac differentiation program (e.g. expression of RYR2, TNNT2, ACTN2 genes, Fig. 1B) in the fused cells. However, despite the robust expression of both rat and human cardiac genes, sarcomeric structures in hybrid cells gradually disassembled (Fig. 6C&D, Supplementary Fig. S9) leading to the loss of contractile function. Molecular mechanisms of this contractility loss remain to be elucidated, but could involve hMSC-induced epigenetic de-differentiation of NRVM genome in fused cells^10^. This same process may be responsible for lower total L-type Ca^2+^ currents produced by hybrid cells compared to NRVMs (Fig. 5D). While revealing, these findings need to be interpreted in light of the simplified nature of our *in vitro* system (e.g. use of healthy neonatal vs. injured adult cardiomyocytes, 2D vs. 3D environment, no inflammatory cells or factors, basic extracellular matrix). It is possible that within the complex biochemical and biophysical environment of the injured heart, hybrid cells would be able to maintain their contractility and directly benefit cardiac function.

Previously, partial fusion between hMSCs and cardiomyocytes has been reported to involve the formation of mitochondria-trafficking nanotubes^9^,^10^,^11^, while mechanisms of permanent fusion attributed to α4β1 integrin/VCAM-1 were only studied in co-cultures of HL-1 mouse CM line and human CD34^+^ hematopoietic cells^16^. In our study, permanent fusion of hMSCs and NRVMs was reversibly blocked by different inhibitors of L-type Ca^2+^ channels and myosin II activity. Complete reversibility of the fusion efficiency (Fig. 7A) ruled out the potential toxicity of the drugs and suggested their direct interference with the fusion process. As all of these drugs also inhibited contractions of NRVMs, we tested the involvement of stretch-activated channels by application of their broad inhibitors, gadolinium^42^ and streptomycin^43^, but found no interference with the fusion process (Supplementary Fig. S10). Alternatively, given the Ca^2+^-dependence of certain fusion proteins such as synaptobrevin (involved in the SNARE fusion complex)^44^, it is possible that decreased intracellular Ca^2+^ concentration following L-type Ca^2+^ channel inhibition may have altered SNARE activity and prevented fusion. However, attempts to vary the intracellular Ca^2+^ concentration by extracellular application of EDTA or CaCl_2_ caused either detachment of hMSCs and NRVMs (EDTA) or Ca^2+^ overload and toxic effects on NRVMs (CaCl_2_). Additionally, application of α-adrenergic (phenylephrine) or β-adrenergic (isoproterenol) receptor agonists to alter intracellular Ca^2+^ handling in NRVMs had no effect on the fusion process (Supplementary Fig. S10). Moreover, despite preventing cell fusion, myosin II inhibitors in our study (and those by others^45^) did not affect Ca^2+^ transients in NRVMs, suggesting that intracellular Ca^2+^ oscillations were not critical for the hMSC-NRVM fusion. Instead, it is possible that a specific combination of biophysical states of the hMSC and NRVM cell membranes within the initial 12 hr window of co-culture (Fig. 6A) is required to engage the cell actomyosin mechanotransduction system^46^, triggering fusion. Indeed, a balance between membrane rigidity and receptor-based signaling was recently found to be crucial for the process of phagocytosis^47^, and it is foreseeable that fusion may involve similar interactions between cellular membranes. Certainly, future studies will be needed to fully understand the underlying mechanisms of the fusion process.

From an electrophysiological standpoint, the hybrid cells described in this study exhibit an intermediate functional phenotype between non-fused hMSCs and NRVMs, both with respect to action potential parameters (upstroke velocity, resting potential) and Ca^2+^ currents. The presence of voltage oscillations in the non-fused hMSCs (Figs 1D and 5A) shows their ability to electrotonically couple with NRVMs, which may have pro-arrhythmic consequences in cell therapy applications, previously suggested *in vitro*^48^. A more cardiac-like electrophysiological phenotype of the hybrid cells and their improved ability to couple with cardiomyocytes compared to non-fused hMSCs may also reduce these arrhythmogenic risks by better supporting action potential conduction in a setting of cardiac injury. Furthermore, the lack of contractile phenotype in hybrid cells suggests that despite potential electrophysiological benefits for the injured heart, it may be most preferable to selectively inhibit fusion of host cardiomyocytes with transplanted hMSCs to permit their positive paracrine action while preventing the fusion-induced contractile loss. However, fused hMSCs may also proliferate and re-acquire contractile function to induce cardiac regeneration *in vivo*, similar to repair of retinal neurons after fusion with bone marrow cells^49^. As such, specific culture conditions to enhance proliferation of the hybrid cells and differentiate them toward a contractile cardiac phenotype warrant further investigations.

In summary, using a variety of structural and functional labeling strategies we have shown that a large fraction of hMSCs rapidly fuses with NRVMs in a 2D *in vitro* co-culture system to generate hybrid cells that posses some but not all cardiomyocyte-like properties. The fusion process is dependent on actomyosin interactions and does not seem to be influenced by cell motility or intracellular Ca^2+^ cycling. Importantly, while the hybrid cells are electromechanically coupled to nearby NRVMs, they lack replicative or contractile behavior needed for immediate utility in cardiac cell therapies. Still, the evidence of post-fusion activation of human cardiac gene program and favorable electrophysiological properties warrant future studies in animal models of cardiac repair.

## Additional Information

**How to cite this article**: Shadrin, I.Y. *et al.* Rapid fusion between mesenchymal stem cells and cardiomyocytes yields electrically active, non-contractile hybrid cells. *Sci. Rep.* **5**, 12043; doi: 10.1038/srep12043 (2015).

## Supplementary Material

Supplementary Information

Supplementary Video 1

Supplementary Video 2

Supplementary Video 3

Supplementary Video 4

Supplementary Video 5
