# Supplementary material for: Rapid fusion between mesenchymal stem cells and cardiomyocytes yields electrically active, non-contractile hybrid cells
Source: Sci Rep. 2015 Jul 10;5:12043. doi: 10.1038/srep12043 (PMC4498233; doi:10.1038/srep12043)
Supplement: Supplementary Information [file srep12043-s1.pdf]

Rapid fusion between mesenchymal stem cells and cardiomyocytes yields electrically active, non-contractile hybrid cells

Ilya Y. Shadrin[1#], Woohyun Yoon[1#], Liqing Li[1], Neal Shepherd[1], and Nenad Bursac[1*]

[1]*Department of Biomedical Engineering, Duke University, Durham, NC*

[#]I.S. and W.Y. contributed equally to this work

*Corresponding author:

Nenad Bursac, PhD

Associate Professor of Biomedical Engineering

Faculty of Cardiology

Duke University

3000 Science Drive

Hudson Hall, Room 136

Durham, NC 27708

phone: 919-660-5510

fax: 919-684-4488

e-mail: nbursac@duke.edu

Supplementary Methods/Materials

Supplementary Methods

Generation of lentiviruses

Several lentiviral vectors were produced using 293T cells. Specifically, different reporter genes including eGFP, gCaMP3, mCherry, gCaMP3-H2B-eGFP, H2B-mCherry, LoxP-mCherry-STOP-LoxP-eGFP, and Cre recombinase were cloned into a pRRL-CMV plasmid (Addgene). For lentiviral production, 3 mL of Opti-MEM (Gibco) was combined with 10ug of desired reporter plasmid, 5ug of psPAX2 packaging plasmid (Addgene), and 2ug of pCMV-VSV-G envelope plasmid (Addgene), and 30uL of Lipofectamine2000 (Invitrogen). The Lipofectamine/plasmid was added drop-wise onto 293T cells cultured at confluence at a density of 6-7x10^6 cells/10cm and 6 hrs later replaced with fresh DMEM (Gibco) medium. After 2-3 days, supernatant was harvested and centrifuged at 500g for 10min, filtered through a 0.45um filter, added to Lenti-X concentrator (Clontech, used as 4x), then mixed and incubated overnight at 4ºC. The following day, mixture was centrifuged at 1500g for 45min at 4ºC, and the pellet was resuspended in 1/10 to 1/100th of the original volume using PBS. Prepared viral stocks were stored at -80ºC. All lentivirus-containing materials were bleached and properly disposed of in accordance with National Institutes of Health guidelines.

Cell preparation (continued)

Rat bone marrow-derived MSCs were prepared and characterized as previously described[1] and utilized for co-culture experiments at passages 5-10. Mouse embryonic

stem cell derived cardiomyocytes (mESC-CMs) were differentiated and genetically purified as previously described.[2] Briefly, D3 mESCs (ATCC CRL-11632) were co-transfected by electroporation (Amaxa, A023) with two constructs. The first construct allowed constitutive expression of G418 resistance under SV40 promoter and puromycin N-acetyl-transferase (Invivogen, pORF39-PAC) expression under a 5.5Kb mouse Myh6 promoter, which was a kind gift from Dr. Jeffrey Robbins.[3] The second construct coded for expression of red fluorescence protein (pDsRed2-1, Clontech) under the same Myh6 promoter. Population of >95% pure mESC-CMs were isolated from suspension cultures of 13-day old differentiating embryoid bodies after 5 days of puromycin selection.

Electrophysiology (continued)

To examine the effect of caffeine on hMSCs during measurement of transmembrane potentials, two large-tipped pipettes (~30 μm) were placed near the edge of a cell, one containing Tyrode's solution with 10 mM caffeine and the other just Tyrode's solution. Drug was applied rapidly to the cell by means of a micrometer-driven syringe connected to the pipette by a solution-filled silicone tube, then removed by a similar application of the control drug-free solution.

For voltage-clamp experiments, cells from co-cultures were first dissociated and re-plated at low density in order to electrically isolate individual cells. Briefly, cells were incubated with prewarmed 0.25% trypsin at 37ºC for 1.5min, washed gently with PBS, and incubated with pre-warmed enzyme mixture containing 1mg/ml collagenase (Sigma, type L), 0.05% trypsin (Worthington, type II), 0.0006 mg/ml DNAase, 1 mg/ml FA-free BSA and 25 μM $CaCl_2$ in a buffered salt solution for 4 min at 37°C. Following very gentle trituration

(3-4 min at 35ºC), the cell suspension was filtered (Celltriks, 50 micron mesh) into 10 ml bicarbonate-buffered saline with 4% horse serum, 2% FBS, 0.029% L-Glutamine and 100 μM $CaCl_2$, then centrifuged for 4.5 min at 1000 rpm. Cells were then re-suspended in 3 ml of the same solution with added $CaCl_2$ to bring Ca^{2+} concentration to 0.5 mM and allowed to rest at 37 ^{o}C for an hour before switching to modified Tyrode's solution. Patch pipets had a resistance of 2 – 5 MΩ when filled with (in mM): $CsMeSO_4$, 130; Mg Gluconate, 2; Cs_2EGTA, 0.02; Succinnate, 5; Oxalacetate, 5; Hepes, 10; Na_2ATP, 5; pH7.3. After establishing a GΩ seal, cells were clamped to a holding potential of -45 mV to inactivate sodium and T-type Ca^{2+} currents before applying a series of voltage pulses between -50 mV and +60 mV. Recorded L-type Ca^{2+} current was filtered at 2 kHz and digitized for storage at 4,000 samples/s (using pClamp8, Axon). Ca^{2+} current amplitudes were quantified as the difference between the inward peak current and the steady state current in each pulse.[4] Cell capacitance was determined by integrating the current in response to a 5 mV hyperpolarizing test pulse. All quantifications were made with custom algorithms in LabTalk (OriginLab).

Immunofluorescence

Immunostainings were performed as previously described[5] using the following primary antibodies: mouse anti-sarcomeric α-actinin (SAA, Invitrogen A10468, Sigma A7811), rabbit polyclonal anti-connexin43 (Cx43, Abcam ab11370), rabbit polyclonal anti-cardiac troponin I (cTnI, Abcam, ab47003), rabbit polyclonal anti-cardiac troponin T (cTnT, Abcam, ab45932), rabbit polyclonal anti-Ki67 (Abcam, ab15580), and mouse monoclonal anti-heavy chain cardiac Myosin (MHC, Abcam, ab15). Non-conjugated primary antibodies

were all used at a 1:200 dilution. Secondary antibodies (Invitrogen Alexa Fluor®) and DAPI were used at a 1:400 dilution. Images were taken on a Zeiss Axio Observer inverted confocal microscope and analyzed with Zeiss LSM 510 software.

Supplementary Video Legends

Supplementary Video 1. Live-cell imaging of 1day-old co-culture of hMSC-gCaMP3 and NRVMs demonstrating synchronous gCaMP3 flashes of a large fraction of hMSCs. Related to Fig. 1A. Scale bar 100μm.

Supplementary Video 2. Cellular fusion observed during a 4.5hr time-lapse video-microscopy of freshly co-cultured hMSC-gCaMP3 with mCherry-labeled NRVMs demonstrating rapid exchange of cytoplasmic contents between NRVM and hMSC, followed by cyclic gCaMP3 flashes and morphing of the membrane contour. Related to Fig. 6B. Scale bar 50μm. Time stamp displayed in hrs:mins.

Supplementary Video 3. Live-cell imaging of early fused cell (<12hr hMSC-gCaMP3/NRVM-mCherry co-culture) showing evidence of contractile function. Video was first taken through a green filter to show a gCaMP3 flash, and then switched to red filter to show contraction of fused cell and surrounding NRVMs. Scale bar 50μm.

Supplementary Video 4. Live-cell imaging of 7d hMSC-gCaMP3/NRVM-mCherry co-culture showing a non-contracting fused cell. Video first shows a still image of a

gCaMP3+/mCherry+ fused cell, followed by gCaMP3 flashes with no evidence of active contractions in the fused cell. Peripheral stretching of fused cell membrane comes from passive pulling by neighboring NRVMs. Scale bar 50μm.

Supplementary Video 5. 4.5hr time-lapse video-microscopy of freshly co-cultured hMSC-gCaMP3 with mCherry-labeled NRVMs showing cellular fusion of a non-motile hMSC. Scale bar 50μm. Time stamp displayed in hrs:mins.

Supplementary References

1. Pedrotty DM, Klinger RY, Badie N, Hinds S, Kardashian A and Bursac N. Structural coupling of cardiomyocytes and noncardiomyocytes: quantitative comparisons using a novel micropatterned cell pair assay. *Am J Physiol Heart Circ Physiol*. 2008;295:H390-400.

2. Liau B, Christoforou N, Leong KW and Bursac N. Pluripotent stem cell-derived cardiac tissue patch with advanced structure and function. *Biomaterials*. 2011;32:9180-7.

3. Subramaniam A, Jones WK, Gulick J, Wert S, Neumann J and Robbins J. Tissue-specific regulation of the alpha-myosin heavy chain gene promoter in transgenic mice. *J Biol Chem*. 1991;266:24613-20.

4. Isenberg G and Klockner U. Calcium currents of isolated bovine ventricular myocytes are fast and of large amplitude. *Pflugers Arch*. 1982;395:30-41.

5. Badie N and Bursac N. Novel micropatterned cardiac cell cultures with realistic ventricular microstructure. *Biophys J*. 2009;96:3873-85.

Species	Gene	Gene Product	Forward Primer	Reverse Primer	Product Size
Human	GAPDH	GAPDH	GTCAGTGGTGGACCTGACCT	AGGGGAGATTCAGTGTGGTG	395
	TNNT2	cTnT	ACCAGGGCAGAAGAAGATGA	CTCTGCCCGACGTCTCTC	279
	ACTN2	SAA	GGCGCTGAAGAAATTGTTG	TGTTCACATTTCTATAAGGAG CAG	166
	RYR2	RyR	GTTGGAGTCGTCCTGGTTGT	TCCTACCCACTTGAGCCACT	297
	GJA1	Cx43	AATTCAGACAAGGCCCACAG	CATGGCTTGATTCCCTGACT	214
	CACNA1C	$Ca_{V1.2}$	GGGAATCCAAGGAGGAGAAG	AGGCGAAACCTGTTGTTAGA	283
	SLC8A1	NCX_1	CTGGGGAAGATGATGACGAC	TGACGTTACCTATGGAGGCG	345
Rat	GAPDH	GAPDH	GGCATTGCTCTCAATGACAA	AGGGAGATGCTCAGTGTTGG	219
	RYR2	RyR	GGAACCTCAGAAATCAAGTA TGGA	AAGTCAACAGTGGGCAGCTT	293

Supplemental Table 1. Species-specific primers for RT-PCR of hMSC-NRVM co-cultures. GAPDH, Glyceraldehyde 3-phosphate dehydrogenase (house-keeping gene). cTnT, cardiac troponin T. SAA, sarcomeric α-actinin. RyR, Ryanodine receptor. Cx43, connexin-43. $Ca_{V1.2}$, L-type Ca^{2+} channel. NCX_1, Na^+/Ca^{2+} exchanger.

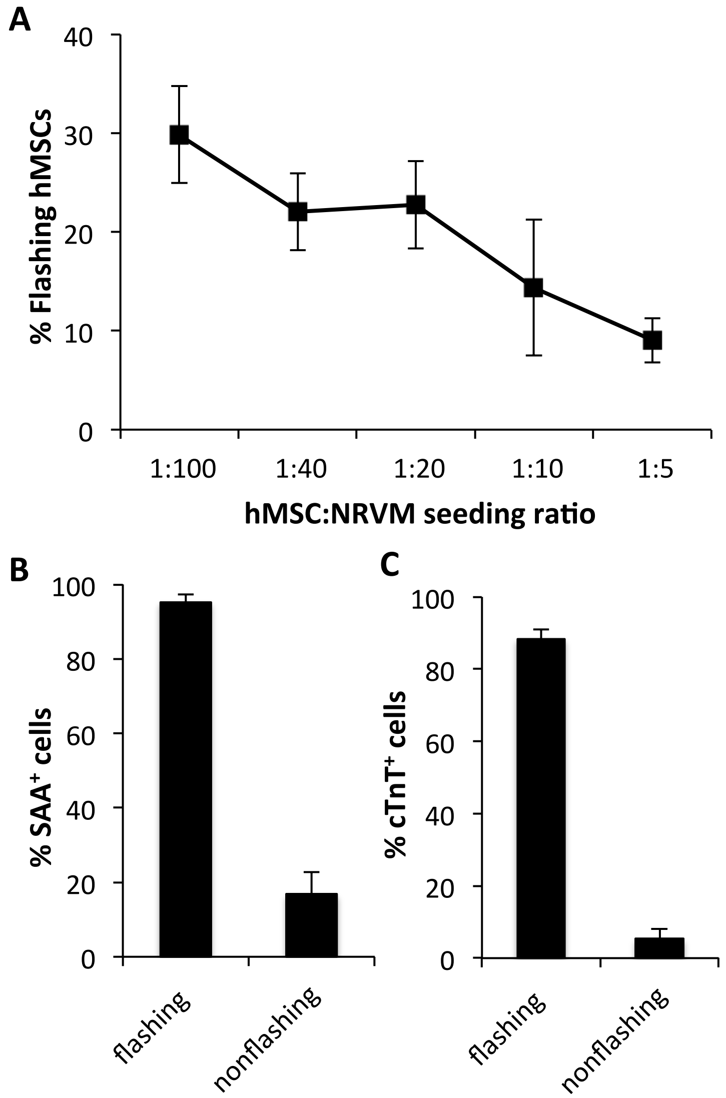

Supplementary Fig. S1. Characteristics of flashing hMSCs in co-culture with NRVMs. A) Percent flashing hMSC-gCaMP3 in 1 day-old co-cultures made with varying hMSC:NRVM seeding ratios; n=3 co-cultures per seeding ratio. B,C) Quantification of immunostaining for sarcomeric α-actinin (SAA, B) and cTnT (C) in hMSC-gCaMP3 cells that were either flashing or non-flashing in live-cell culture; n=3 co-cultures, N=38-92 cells tracked per co-culture.

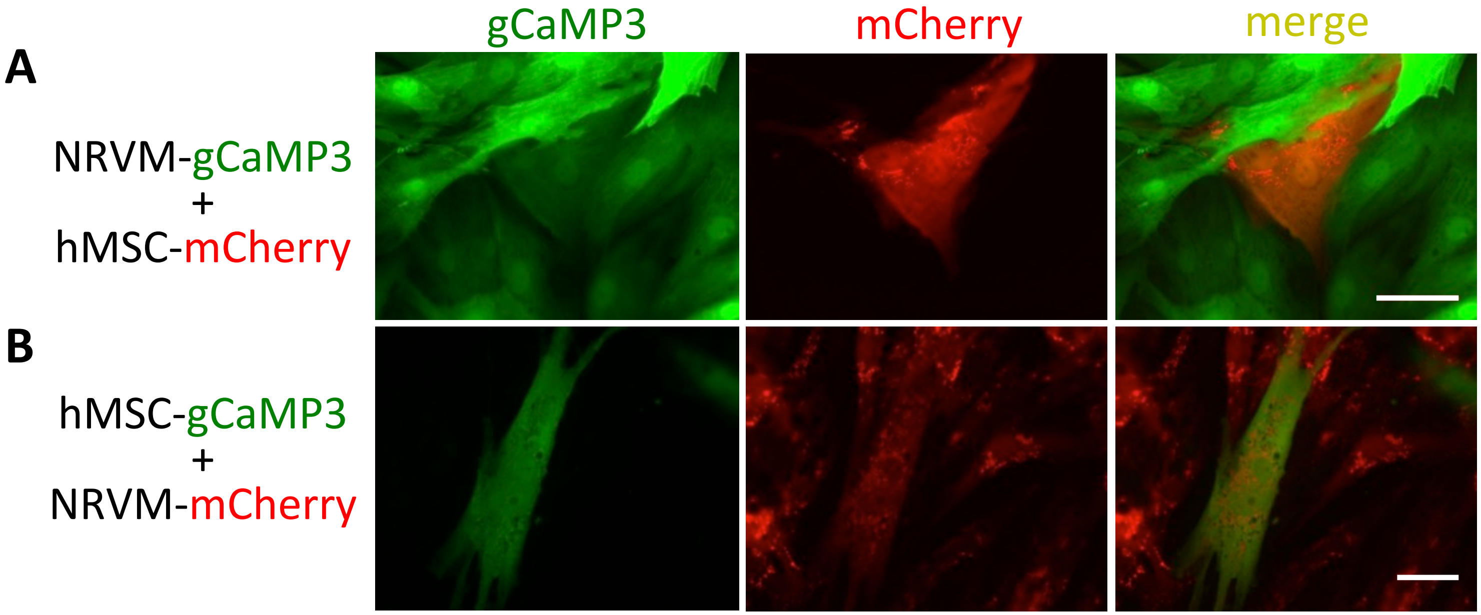

Supplementary Fig. S2. hMSC-NRVM fusion occurs regardless of plating or labeling order. A) Representative images from a 7-day co-culture of NRVM-gCaMP3 with hMSC-mCherry (reverse labeling) show presence of dually-labeled cells indicative of cellular fusion. B) Representative images from a 6-day co-culture of hMSC-gCaMP3 with NRVM-mCherry (reverse plating order, first hMSCs, followed by NRVMs) show evidence of dually-labeled cells indicative of cellular fusion. All scale bars 50 µm.

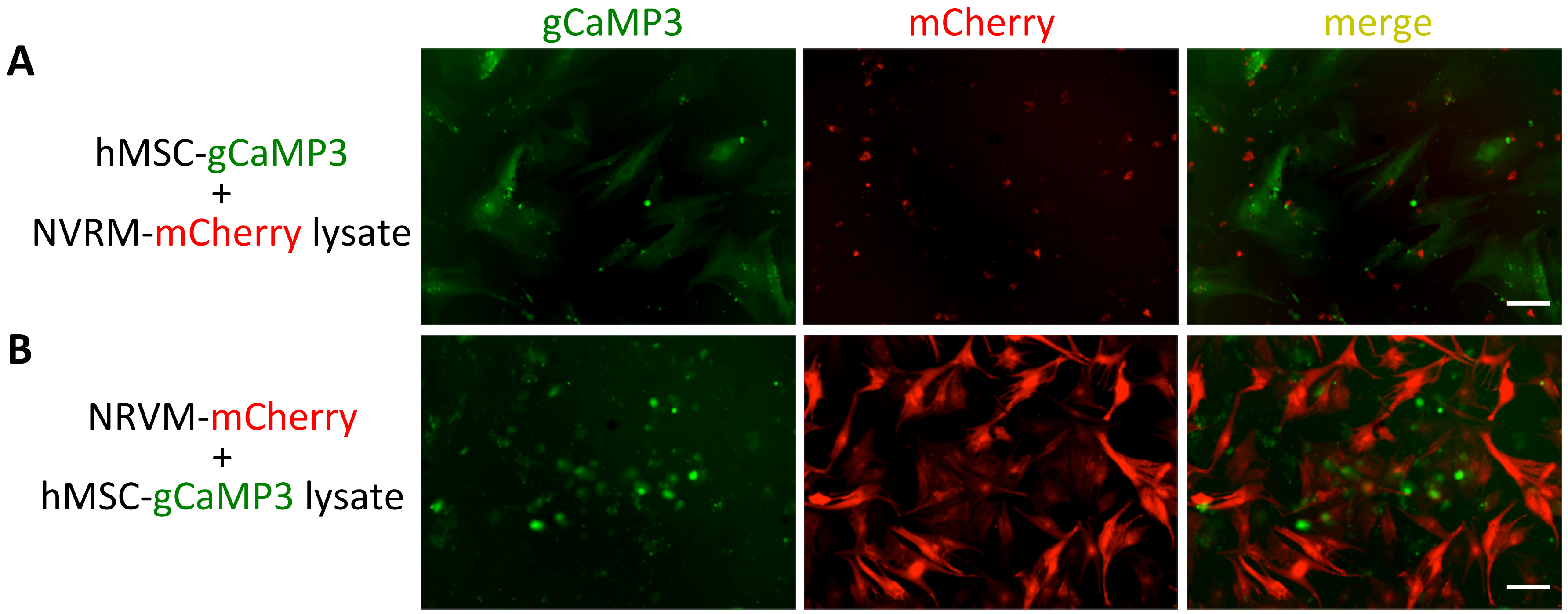

Supplementary Fig. S3. Absence of phagocytosis in hMSC-NRVM co-cultures. A) Representative images from a culture of hMSC-gCaMP3 with added lysate from NRVM-mCherry show no uptake of NRVM lysate in hMSCs. B) Representative images from a culture of NRVM-mCherry with added lysate from hMSC-gCaMP3 show no uptake of hMSC lysate in NRVMs. All scale bars 100 µm.

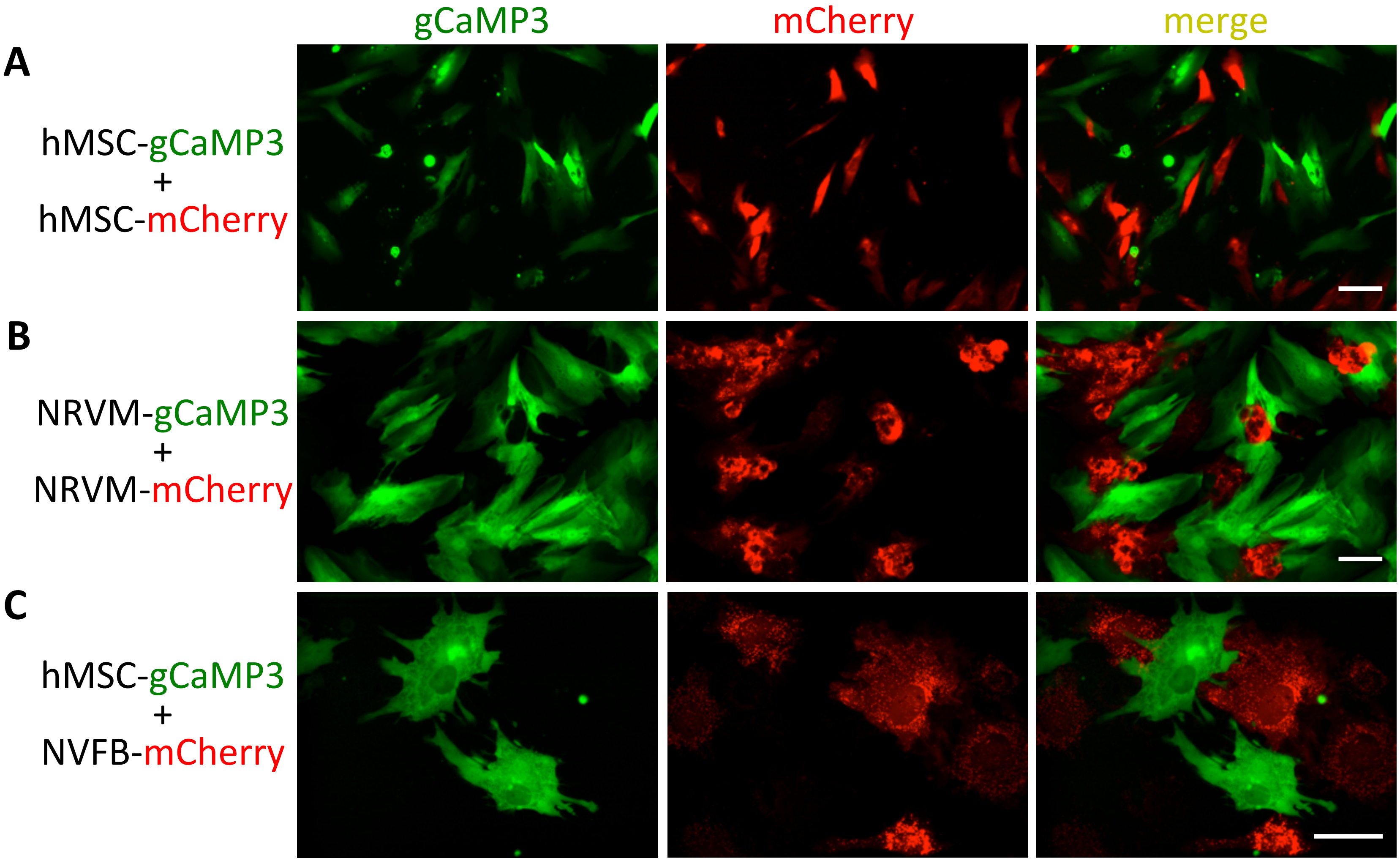

Supplementary Fig. S4. Cell fusion in co-cultures is specific to hMSCs and NRVMs. A) Representative images from a 7-day co-culture of hMSCs labeled with either gCaMP3 or mCherry shows no homocellular fusion between hMSCs. B) Representative images from a 7-day co-culture of NRVMs labeled with either gCaMP3 or mCherry shows no homocellular fusion between NRVMs. C) Representative images from a 7-day co-culture of hMSC-gCaMP3 with neonatal rat ventricuar fibroblasts (NVFB) labeled with mCherry (NVFB-mCherry) shows no fusion between hMSCs and NVFBs. All scale bars 50 µm.

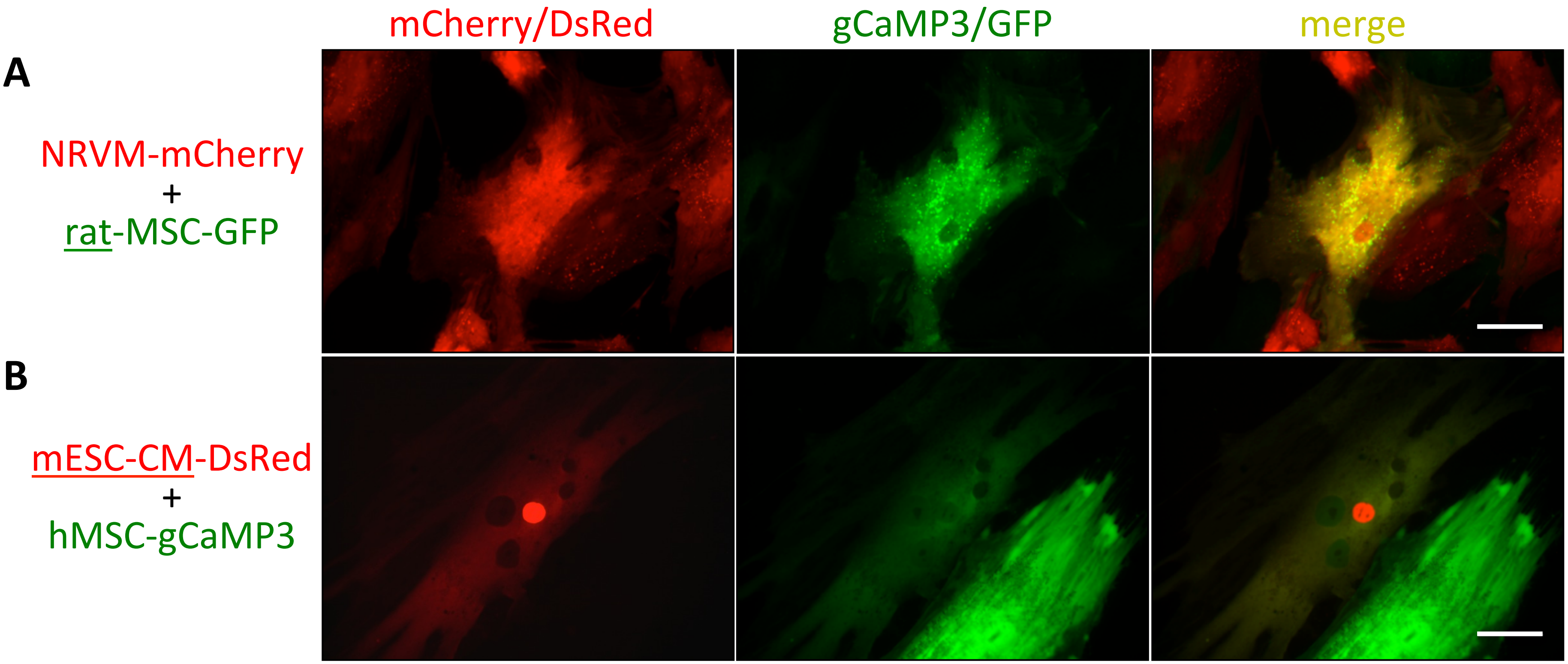

Supplementary Fig. S5. MSC-cardiomyocyte fusion is not species specific. A) Representative live-cell images from a 7-day co-culture of NRVM-mCherry with adult rat MSC-GFP showing rat-rat MSC-cardiomyocyte fusion. B) Representative live cell images from a 7-day co-culture of DsRed labeled mouse-ESC-derived cardiomyocytes (mESC-CM-DsRed) with hMSC-gCaMP3 showing mouse-human MSC-cardiomyocyte fusion. All scale bars 50 µm.

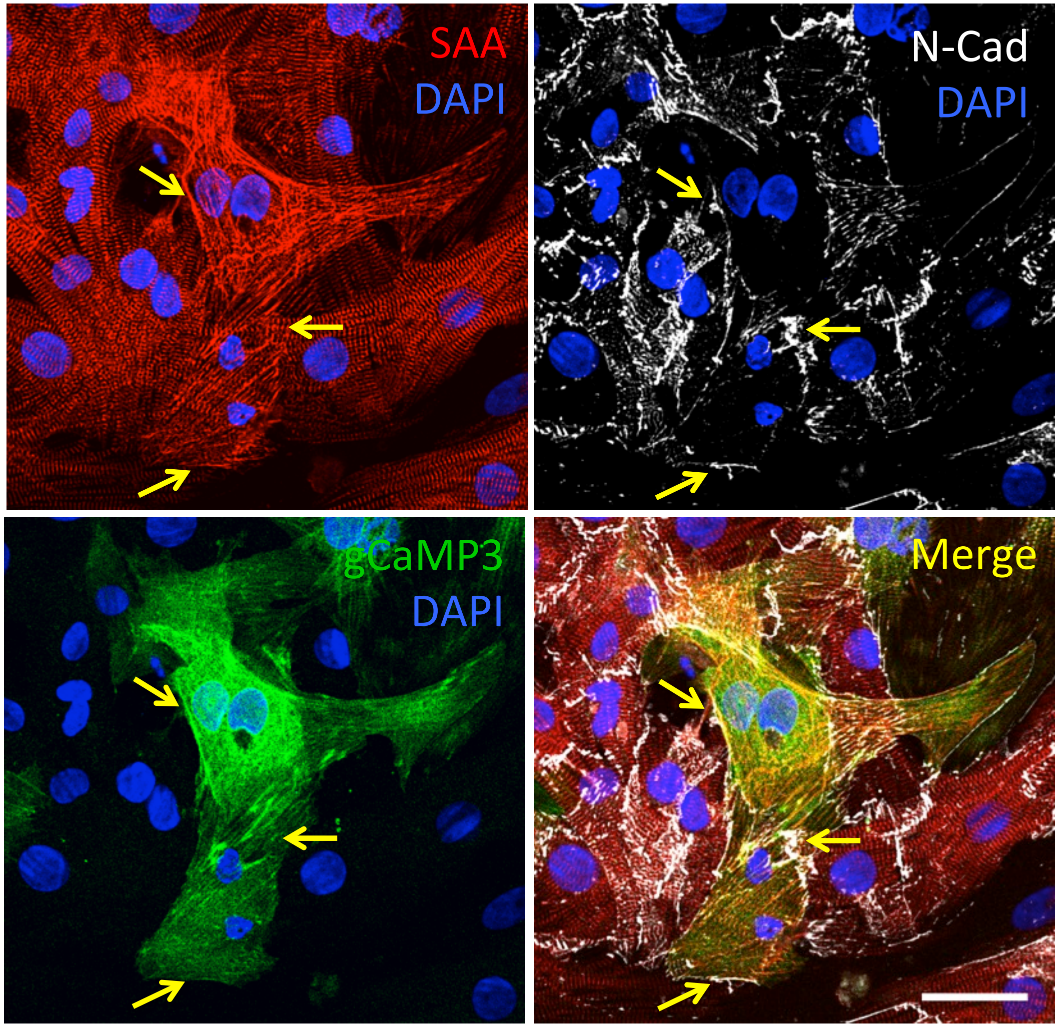

Supplementary Fig. S6. N-cadherin expression in fused cells. Representative confocal images from a 7d-old co-culture of hMSC-gCaMP3 and unlabeled NRVMs. Note presence of N-Cadherin^{+} junctions (arrows) bordering gCaMP3^{+}/SAA^{+} fused hMSCs. Scale bar 50µm.

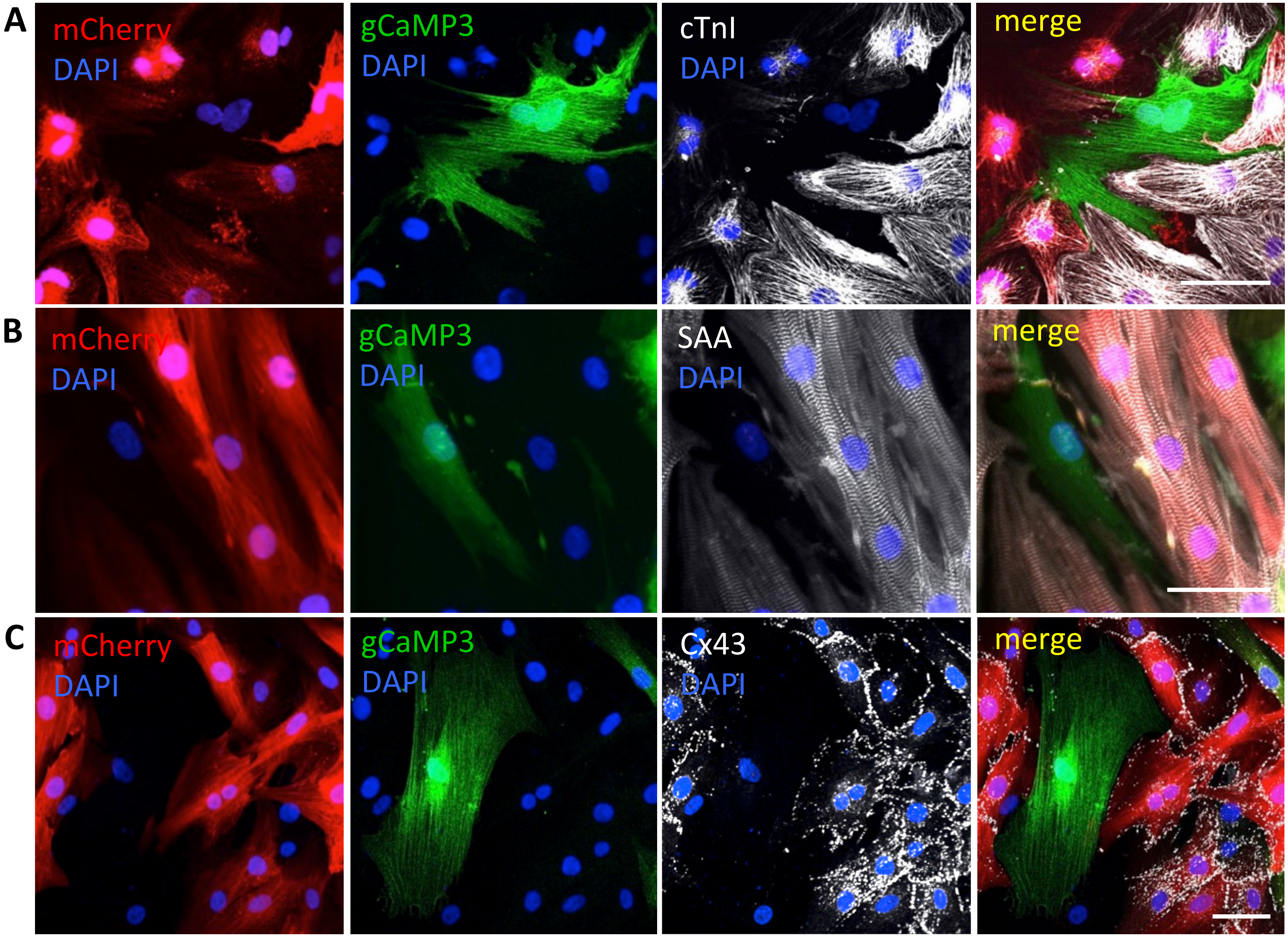

Supplementary Fig. S7. Non-fused hMSCs do not express cardiac markers. A,B) Representative images of hMSC-gCaMP3/NRVM-mCherry co-cultures immunostained for cTnI (A), SAA (B), and Cx43 (C) demonstrating lack of cardiac expression in non-fused (gCaMP3^{+}/mCherry^{-}) hMSCs. All scale bars 50µm.

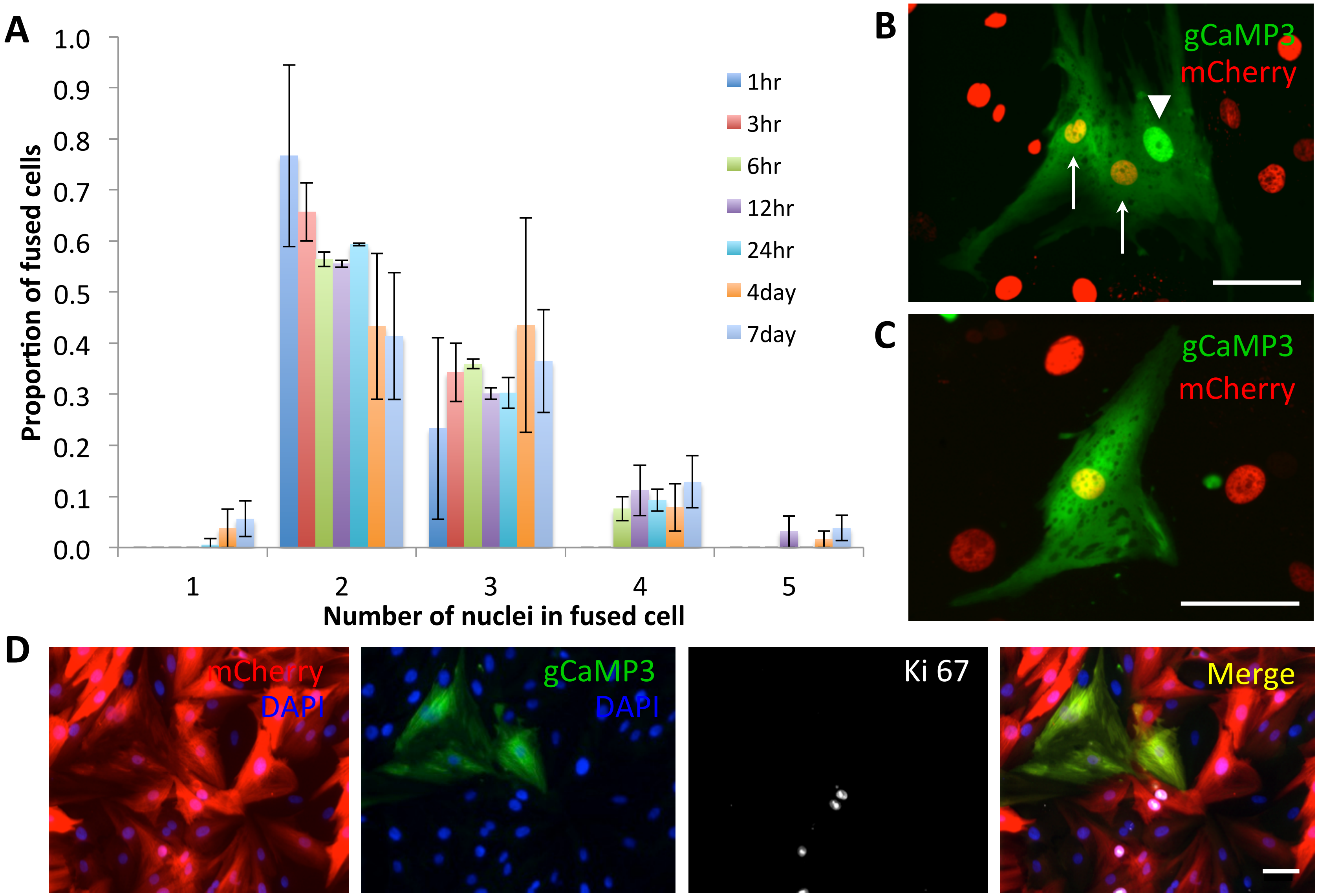

Supplementary Fig. S8. Fused cells are predominantly non-proliferative heterokaryons. A) Nuclear cell number in fused cells at various times during co-culture; n=2-3 co-cultures per time point, N=10-40 cells per co-culture. B-C) Representative fused cells from a 7d-old NRVM-H2B-mCherry/hMSC-gCaMP3 co-culture showing a heterokaryon (B) with 2 NRVM nuclei (arrows) and 1 hMSC nucleus (arrowhead) and a single-nucleated synkaryon (C). D) Representative images of a group of fused cells immunostained for the proliferation marker Ki67 at 7 days of co-culture. No fused cells showed $Ki67^{+}$ staining. All scale bars 50 µm.

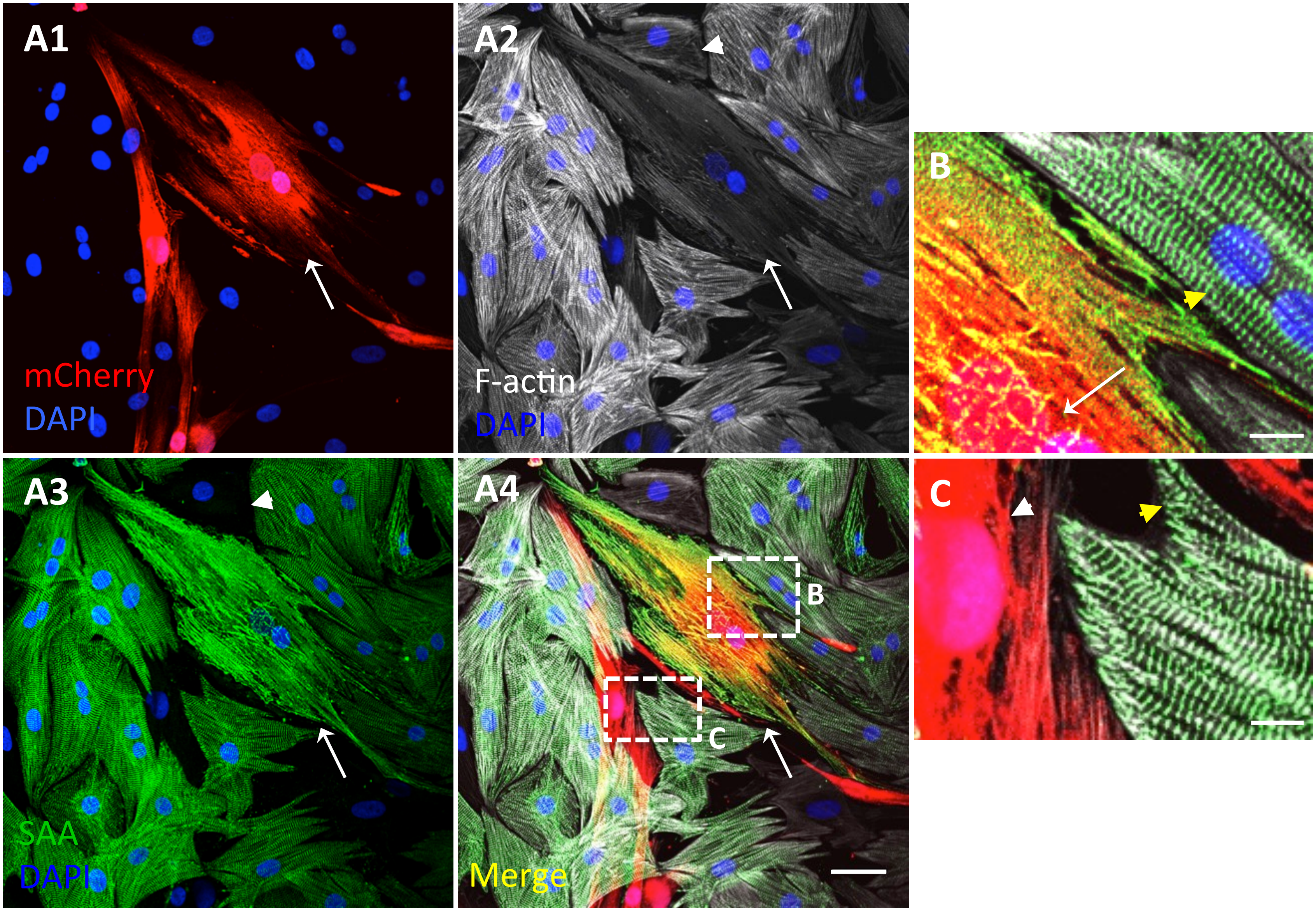

Supplementary Fig. S9. Loss of striations in fused cells. Representative confocal image of 7d-old co-culture of hMSC-mCherry and NRVMs showing loss of striations in fused cell (A-B, arrows). In contrast, neighboring NRVMs express robust cross-striations (B-C, yellow arrowheads). Note the SAA^{-} cardiac fibroblast (A2-3, white arrowhead) and non-fused hMSC (C, white arrowhead). Scale bars 50µm (A1-4), 10µm (B-C).

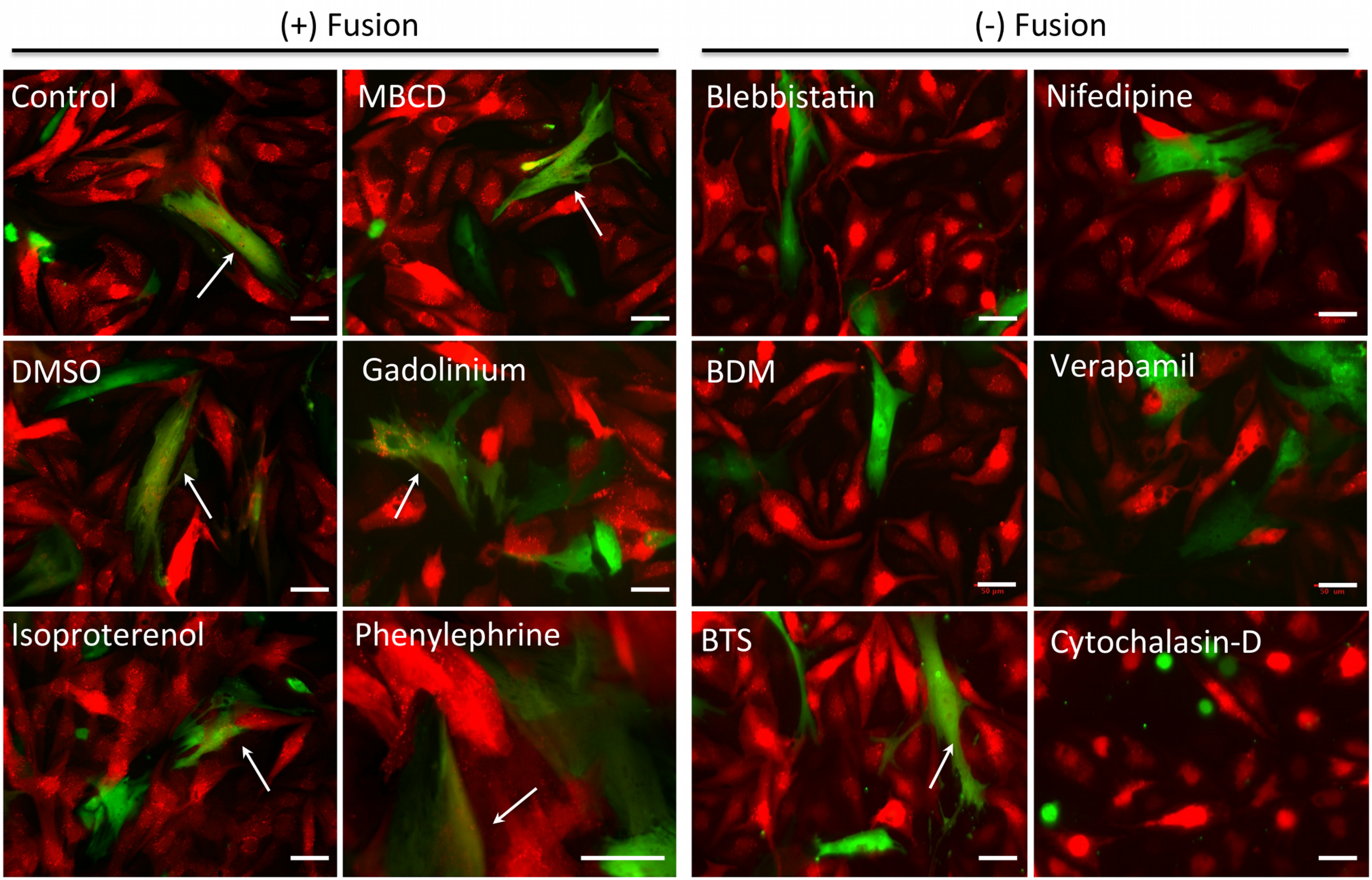

Supplementary Fig. S10. Selective inhibition of hMSC/NRVM fusion. Representative images of 1-day old hMSC-gCaMP3/NRVM-mCherry co-cultures. Fusion is inhibited by myosin II inhibitors (blebbistatin, BDM, and BTS) and L-type Ca2+ inhibitors (Nifedipine and Verapamil) but not by other drugs (MBCD, DMSO, isoproterenol, gadolinium, and phenylephrine). Cytochalasin-D prevented spreading of hMSCs. Fused cells marked with white arrow. Rare fused cells were seen with BTS treatment. All scale bars 50 µm
